# Double vulnerability of active-NRF2 lung squamous cell carcinoma to NRF2 and TRIM24

**DOI:** 10.1186/s12943-025-02401-y

**Published:** 2025-07-17

**Authors:** Miriam Sánchez-Ortega, Antonio Garrido, Lorena Sanz, Rafael Torres-Pérez, Carmen Hernandez, Alvaro Gutierrez-Uzquiza, Ming Sound Tsao, Ana Clara Carrera

**Affiliations:** 1 Department of Immunology and Oncology, Scientific Research Council (CSIC), Centro Nacional de Biotecnología, Autónoma University Madrid, Cantoblanco, Madrid, E-28049 Spain; 2 Nanocaging Research Group, Department of Biosciences, School of Biomedical and Health Sciences, European Univ of Madrid, Villaviciosa de Odón, Madrid, E-28670 Spain; 3 Department of Biochemistry and Molecular Biology, Pharmacy Faculty, Complutense University of Madrid, Madrid, Spain; 4 Princess Margaret Cancer Centre, University Health Network, Toronto, ON Canada

**Keywords:** LUSC treatment; NRF2, TRIM24, CRISPRa/dCas9, PI3Kα

## Abstract

Lung squamous cell cancer (LUSC) is associated with very poor survival due to the lack of specific treatments. A common genetic alteration in LUSC involves mutations in *NFE2L2* (protein named NRF2) or its regulator, *KEAP1*, resulting in increased activity of the NRF2 transcription factor (TF). This study compares the requirement for active-NRF2 in LUSC cell lines. Although normal-NRF2 cells are more sensitive to oxidative stress, they do not require NRF2 for survival under non-stress conditions, in contrast, LUSC cells with active-NRF2 mutations depend on NRF2 for viability. NRF2 depletion in patient-derived organoid cultures with active-NRF2 as well as in xenografts with active-NRF2 triggers cell death. The focus of this study is to find genes that rescue cell death upon NRF2-depletion in active-NRF2 cells. A CRISPRa/dCas9 screening for gene targets capable of rescuing cell survival in these cells identified *TRIM24* as a gene whose expression saves cell survival in NRF2-depleted active-NRF2 LUSC cells. Alongside oxidative stress, the lack of TRIM24 selectively contributed to the induction of cell death (apoptosis and ferroptosis) in active-NRF2 LUSC cells. Cells with a high *NFE2L2/KEAP1* copy number ratio also undergo cell death. The increase in cell death observed upon TRIM24 depletion involves a reduction of TRIM24/PI3Kα complexes which destabilizes the PI3Kα catalytic subunit. Notably, overexpression of PI3Kα rescues cell survival in TRIM24-depleted active-NRF2 cells. These findings point to novel therapeutic approaches in LUSC.

**Supplementary Information:**

The online version contains supplementary material available at 10.1186/s12943-025-02401-y.

## Introduction

Lung cancer is the most lethal malignancy worldwide, causing over 1.6 million deaths yearly [1]. The second most commonly diagnosed cancer in 2020, it has the lowest 5-year survival rate among high-incidence malignancies such as breast, colorectal, prostate, skin, etc. cancer. Lung cancer has been divided into 2 groups: small-cell lung cancer, representing 15% of cases, and non-small cell lung cancer (NSCLC) (85%). The latter includes adenocarcinoma (LUAD), squamous cell carcinoma (LUSC), large-cell carcinoma and other less frequent types. LUSC tumors are composed of polygonal cells with squamous differentiation, located in the proximal part of the bronchial surface. No specific therapy exists for LUSC, and its poor prognosis makes it essential to establish new approaches.

Novel cancer-treatment strategies involve targeted therapies that use compounds or antibodies inhibiting specific signaling pathways required for tumor progression [2]. While therapeutic targets have been identified for NSCLC, these advances have mostly benefited patients with LUAD, since individuals with LUSC do not meet the overall response rate and progression-free survival required to be clinically beneficial [3].

The Cancer Genome Atlas describes a set of genes frequently mutated in LUSC, including *TP53*,* CDKN2A*,* PTEN*,* PIK3CA*,* KEAP1*,* NFE2L2*,* NOTCH1*, and *RB1* [4, 5]. Signal transduction pathways involving these genes include the phosphoinositide 3-kinase (PI3K) pathway (mutations in *PTEN* and *PIK3CA*) and the redox response signaling pathway (*NFE2L2* and *KEAP1*), and others [6]. Among them, *NFE2L2* encodes NRF2, the master transcription factor (TF) for cell protection against reactive oxygen species (ROS). Although ROS is needed for certain physiological cell responses, an excess of these radicals causes oxidative damage in DNA, lipids, and proteins, triggering cell death. NRF2 interacts with antioxidant response element (ARE), forming a heterodimer with one of the 3 small MAFs (sMAF) [4, 5]. NRF2 activity is negatively controlled mainly by *KEAP1* (Kelch-like ECH-associated protein 1), which regulates NRF2 degradation [7].

Most *NFE2L2* alterations found in LUSC represent gain-of-function mutations, which render NRF2 active, whereas many *KEAP1* mutations render KEAP1 unable to bind to NRF2 and represent loss-of-function mutations [8, 9]. Some *KEAP1* mutations contribute to malignancy independently of NRF2, most likely this is due to additional KEAP1 interactors 1^1^ Additional epigenetic and non-epigenetic mechanisms control NRF2 activation [8, 9]. Although Nrf2 knockout mice exhibit an increased number of lung metastases, suggesting the role of Nrf2 as a tumor suppressor [10], the studies in humans link high NRF2 levels to cancer progression [11–12]. NRF2 regulation of tumor progression could be linked to the hallmark regulators of cancer, most of which are regulated by NRF2 [13]. This is particularly clear in LUSC, which exhibits maximal NRF2 activity [14]. Here, LUSC cell lines with *NFE2L2* or *KEAP1* genetic alterations have been studied to identify potential therapeutic approaches. We examined normal-NRF2 (cells with WT *NFE2L2* and WT *KEAP1*) or global-active-NRF2 cells (with an *NFE2L2* mutation that induces dissociation from KEAP1, or alternatively, with a *KEAP1* mutation that interferes with NRF2/KEAP1 association or with a high *NFE2L2/ KEAP1* copy nº ratio) [15, 16].

Active-NRF2 LUSCs exhibited a greater dependency on NRF2 than those with wild-type, or “normal”- NRF2. Indeed, cells expressing active-NRF2, such as HCC15 cells that have a *KEAP1* p.304G > C mutation underwent cell death upon NRF2 depletion and treatment with ROS. The NRF2 target genes utilized in this study were previously described as NRF2 targets expressed in LUSC [14, 17]. A guide(g)-RNA CRISPRa/dCas9 library was used to induce gene overexpression by targeting gene promoters. This screening revealed that certain sgRNAs amplify genes that rescue cells from death, identifying potential new therapeutic targets for NRF2-active LUSC.

One of the targets identified was tripartite motif 24 protein (TRIM24). TRIM24 functions as an E3-ubiquitin ligase for p53, as a coactivator of the estrogen receptor and as repressor of retinoic acid receptor transcriptional activity [18]. TRIM24 overexpression has been associated with poor overall survival in LUSC patients [19]. Here, we show that TRIM24 expression alone was capable of cell-survival rescue in NRF2-depleted, ROS-treated active-NRF2 LUSC cells. Examination of TRIM24-regulated cell-survival pathways identified PI3K activation as a key mechanism. Although in glioblastoma, KAT6A controls *PIK3CA* gene expression through TRIM24 binding to this gene promoter [20], in this study, TRIM24 promoted cell survival by associating to PI3Kα protein, thereby increasing its stability. Indeed, PI3Kα stabilization or overexpression rescued cell from death induced by TRIM24 depletion. These findings illustrate the cooperation between survival pathways (NRF2 and TRIM24) in LUSC progression. The data supports the potential use of TRIM24-interfering compounds to destabilize PI3Kα and treat active-NRF2 LUSC patients.

## Materials and methods

### Antibodies and reagents

The antibodies used in Western blotting (WB) were as follows: anti-NRF2 XP Rabbit mAb, anti-PTEN Rabbit, anti-phospho-Akt (Thr308) Rabbit mAb, anti-Akt, anti-AKT substrate Rabbit (the anti-AKT substrate Ab recognizes AKT-phosphorylated proteins), anti-PI3Kα Rabbit mAb, and anti-cleaved Caspase 3 Rabbit mAb (Cell Signaling). Additional antibodies included recombinant anti-NRF2 antibody ChIP grade (Abcam), anti-Calpain 1 (Abcam), anti-KEAP1 Rabbit Polyclonal (Proteintech), antiβ-actin (Sigma), anti-TIF1 Alpha/TRIM24 Rabbit and anti-BRD7 Rabbit mAb (both from Fortis Life Sciences), anti-MKRN1 polyclonal (Thermofisher), anti-LC3B (NOVUS), and anti-Ubiquitin (P4D1) (Santa Cruz Biotechnology). For immunofluorescence, the following antibodies were used: anti-NQO1 (A-5) Mouse mAb, anti-NRF2 (H-300) Rabbit Polyclonal (Santa Cruz Biotechnology), Alexa Fluor 555 goat anti-mouse IgG, and Alexa Fluor 488 Goat Anti-Rabbit IgG (Invitrogen). The antibody used for immunostaining was PE anti-human CD184 (CXCR4) (BioLegend). The ROS inducers used were erastin (XC^−^ system inhibitor) and RSL3 (GPX4 inhibitor) (Sigma). NRF2 inhibitors used were AEM1 and ML385 (Sigma). Other reagents used were as follows: geneticin (Calbiochem); puromycin, blasticidin, hygromycin, doxycycline, ampicillin, staurosporine, cycloheximide, carbenicillin, and necrostatin (Sigma); ZVAD and ferrostatin 1 (Abcam); PD 150,606 (Tocris); and MG132 (Sigma) and ALLM (Calpain inhibitor II) (MedChem Express).

### Cell lines, shNFE2L2 inducible, NRF2 depletion, and *PIK3CA* overexpression

The following LUSC cell lines were purchased from the American Type Culture Collection: SK-MES-1 (HTB-58), NCI-H520 (HTB182), and NCI-H226 (CRL-5826). Additionally, HCC-15 (ACC-496) was obtained from the DSMZ collection. Normal airway epithelial cells (HSAEC1/KT) were acquired from ATCC. HEK-293T cells (CRL-3216) were used for virus production. Jurkat cells were kindly provided by Dr. Mario Mellado (National Center for Biotechnology (CNB), CSIC). Cells were maintained in DMEM, RPMI-1640, or MEM (Gibco, Thermofisher Scientific) containing 10% fetal bovine serum (TicoEurope), 2 mM L-glutamine, 10 mM HEPES, 100 IU/ml penicillin, 100 µg/ml streptomycin, 1 mM sodium pyruvate, 100× MEM non-essential amino acids (Biowest), and 50 µg/ml gentamicin (Sigma). Cells were maintained at 37 °C, 5% CO2, and 95% humidity. HSAEK1/KT cells were cultured in SABM™ small airway epithelial cell growth basal medium supplemented with SAGM™ Medium SingleQuotsTM (Lonza). Cells were used in passages 3 to 12 and routinely tested for mycoplasma contamination (MycoAlert Mycoplasma Detection Kit, Lonza). LUSC cells with inducible silencing of the *NFE2L2* gene were generated using lentiviral particles containing the appropriate plasmids. The inducible *NFE2L2* silencing vector was previously designed following the instructions in the pLKO-Tet-On User Manual (Novartis, Addgene). The oligonucleotides designed are described in Supplementary Table 1.

For infection, HEK-293T cells were transfected with jetPEI DNA reagent (Polyplus) and 150 mM NaCl solution, using the Tet-pLKO-neo sh*NFE2L2* vector, along with the pCM-V-VSV-G and psPAX2 plasmids (Addgene) following the manufacturer’s instructions. In parallel, target cells were seeded (150,000 cells/well in a 6-well plate). At 48 and 72 h, supernatants containing viral particles were filtered (0.45 μm; Millipore), supplemented with polybrene (8 µg/ml, Sigma), and added to the target cells. Plates were centrifuged for 90 min at 530 × g. After 5–6 h of incubation, the medium was replaced with the standard culture medium of the target cells. The following day, geneticin (G418, 0.3 mg/ml) was added to the culture medium for selection, and cells were maintained under selection for 15 days. Serial dilutions were performed to isolate clones. The efficiency of infections was screened by WB. Doxycycline (2 µg/ml, 96 h) was added. For the experiments with oxidative stress, the ROS inducer RSL3 (4.5 µM) or Erastin (10 µM ) or their vehicle was added for the last 24 h. In the case of *PIK3CA* overexpression, infection was performed as described above, except that HEK-293T cells were transfected with pLV-SFFV-*PIK3CA*-WPRE-Ubc-Emerald (Addgene 203797). Infected cells were sorted for GFP + cells and tested in WB and RT-qPCR.

### PDX-derived organoids and generation of organoids with *NFE2L2* shrna/sirnas

A xenograft-derived organoid (XDO344) from a lung adenocarcinoma, and 2 XDOs from lung squamous cell carcinoma long-term organoids (XDO377 and XDO274) were established from patient-derived xenografts (PDX) generated from LUAD and LUSC at the University Health Network [21]. The cultures were maintained as described [21]. These organoids were maintained in advanced DMEM/F-12 medium (Gibco) supplemented with 2 mM GlutaMAX, 10 mM HEPES, 100 U/ml Antibiotic-Antimycotic, 1X B-27 Supplement (Gibco), 1.25 mM N-Acetyl-L-cysteine, 100 nM SAG (Sigma), 50 ng/ml recombinant Human EGF (Corning), 40 ng/ml recombinant Human FGF-7, 100 ng/ml recombinant Human FGF-4, 100 ng/ml recombinant Human Noggin (Peprotech), 0.5 µM A 83 − 01, 250 nM CHIR 99,021 (Tocris), and 10 µM Y-27,632 (Selleck Chemicals) in growth factor-reduced Matrigel (Corning). Cells were dissociated into single cells using TrypLE (Gibco) and were passaged every 6–10 days. XDO344-LUAD, and the two LUSC XDO377 and XDO274 were different. Any of the three had *KEAP1* mutations, but the three had *KEAP1* low copy number (-1). As for *NFE2L2*, XDO344-LUAD was normal, XDO377-LUSC was normal and XDO274-LUSC a slight *NFE2L2* copy deletion. XDO274 also showed a *CUL3* p.R305C mutation [with a variant allele fraction in cancer specimens of 57 in 126, near 50%]. The R305C mutation location is in a hot spot in CUL3 in Lung Cancer [22].

XDO377 organoids expressing inducible shRNA for *NFE2L2* were generated using lentiviral particles. Inducible *NFE2L2* silencing vector was previously designed following pLKO-Tet-On. For infection, HEK-293T cells were transfected with Fugene reagent (Promega) together with OPTIMEN solution and Tet-pLKO-neo sh*NFE2L2* vector, plus pCM-V-VSV-G, and psPAX2 plasmids following the manufacturer’s instructions. After 16 h, HEK-293T medium was changed to ISCOVE’s medium plus 30% FBS (Gibco). 48 h later, lentivirus supernatant was passed through a 0.45-µm filter and concentrated 10-fold using Lenti-X-Concentrator (Takara). Single-cell suspensions of XDO377 (375,000 cells in 750 µl of medium) were infected with 45 µl of viral supernatant + 8 µg/ml of polybrene. Each tube was centrifuged at 600 × g (1 h, 32 ºC) and incubated at 37 ºC for 1 h. After centrifugation, 80,000 cells per well were seeded together with Matrigel as described [23]. After 24 h, G418 was added (0.1 mg/ml, 10 days). The efficiency of infections was assessed by qRT-PCR and immunofluorescence (IF) after treatment with doxycycline (2 µg/ml) every 72 h for 15 days.

XDO organoids were dissociated into single cells before transfection. For each 24-well/condition, 100,000 XDO single cells were suspended in 450 µl of medium (Opti-MEM, Gibco) and transfected once with 3.3 µl of siRNA *TRIM24* (20 µM)(see below) mixed with 1.3 µl of lipofectamine RNAimax according to the manufacturer’s instructions. For optimization of transfection efficiency, single-cell transfection was also performed with Opti-MEM plus lipofectamine 2000 (Invitrogen) or advanced DMEM/F-12 medium supplemented with 10% FCS, both with siRNA. Each tube was then centrifuged at 530 × g for 1 h and incubated at 37 ºC for 4–6 h. After centrifugation, 100,000 cells per well were seeded together with Matrigel. After 96 h, organoids were dissociated and collected for qRT-PCR and flow cytometry assays. Only 50% of the organoids successfully incorporated siRNA.

### SiRNA transfection in lung cells

Lung cells were transfected twice (at 24 h and 96 h) with human siRNA for *TRIM24* (Ambion TRIM24 siRNA Cat#4390825), or alternatively, siRNA ON-target Plus SMART pool for *TRIM24* and *MKRN1* (Dharmacon), or Stealth siRNA negative control, low GC (Invitrogen), all at a final concentration of 20 µM. In the case of SKMES-1 and H520, they were transfected once with siRNA ON-target Plus SMART pool *NFE2L2* (20 µM) (Dharmacon) (96 h). Transfections were performed using lipofectamine RNAimax (Invitrogen) and Opti-MEM medium (Gibco) according to manufacturer instructions. Before the second transfection, 150,000 to 300,000 cells were seeded into 6-well plates and after 72 h (hours), cells were treated with RSL3 (4.5 µM) or DMSO (24 h). Cells were collected for WB, qRT-PCR, and propidium iodide (PI) assays. In some experiments, MG132 (Sigma) was added the last 5 h (10µM) before collection. For calpain inhibitor assays, PD 150,606 (Tocris) was added at indicated concentration for 24 h.

### Real-time quantitative PCR (RT-qPCR)

Total RNA was extracted using RNeasy kit (Quiagen). Regarding tumor xenografts, total tissue RNA was extracted using TrizolTM (Invitrogen). Subsequently, 1 µg of total RNA was reverse transcribed with the High-Capacity cDNA Reverse Transcription Kit (Applied Biosystems). qPCR was performed using 5× HOT FIREPol EvaGreen qPCR Mix Plus (ROX) (Solis BioDyne) on the QuantStudio 5 Real-Time PCR System program (Thermo Fisher Scientific). Specific human qPCR primers are listed in Supplementary Table 1. Results were analyzed using *GAPDH*,* β-ACTIN*, or *TBP* as housekeeping genes and the RT value.

### Viability, apoptosis/ferroptosis, CXCR4 staining, western blot, immunoprecipitation and immunofluorescence

Cell viability was analyzed by flow cytometry as previously reported [23] using the probe PI (propidium iodide, Beckman). Cells were centrifuged at 150×g for 5 min and resuspended in staining buffer (PBS + 10% FBS). Cell viability was measured as the proportion of PI-negative cells (10 µl). For apoptosis detection, cells were stained with Annexin V-FITC (Southern Biotech). After washing the cells twice with PBS, cell pellets were incubated with Annexin V-FITC antibody for 15 min in the dark, resuspended in PBS, and analyzed using a Cytomics FC 500 Flow Cytometer 1 L (Beckman Coulter). The percentage of viable cells was calculated based on 10,000 to 30,000 events using Kaluza software (Beckman Coulter; Life Sciences). Alternatively, flow cytometry could be fixed at 60 s per sample, and the number of dead and viable cells was counted. Ferroptosis was assessed as previously described using BODIPY 581/591 C11 (Invitrogen) [23]. Fluorescence intensity was measured in CytoFLEX S (Beckman), analyzing the percentage of PE-A positive cells (Kaluza software). Cell pellets were incubated with anti-CXCR4 antibody (20 min), centrifuged, and resuspended in PBS. CXCR4 staining was measured in Cytomics FC 500 Flow Cytometer 1 L. Mean fluorescence intensity and the percentage of positive cells were recorded for 10,000 events. WB was as described [23].

For immunofluorescence, 100,000 single cells from XDO organoids were centrifuged and fixed in 4% PFA (10 min). Cells were then permeabilized with 0.1% TX-100 in PBS for 30 min. Later, 2.5% BSA and 0.1% TX-100 in PBS was added (30 min). Primary and secondary antibodies were each added for 1 h (1:50 or 1:100, respectively) and DAPI (BIO-RAD) was added for 5 min at 4 µg/ml in PBS with 4% TX-100. Slides were mounted in appropriate medium (Vector laboratories), and images were captured using a Zeiss LSM700 confocal microscope at 20× and 63× magnification. Cell counts with cytoplasmic or nuclear NRF2 staining were analyzed using ImageJ software (NIH).

For immunoprecipitation (IP), cells were collected in PBS plus NEM (1 µM). Additionally, NEM (100 µM), MG132 (5 µM), and PR-619 (50 µM) were added to RIPA or association buffers. For protein-protein association buffer was used (20mM Tris-HCl, pH 8.0, 137 mM NaCl, 1 mM MgCl2, 1 mM CaCl2,1% NP-40); both lysis bffers containing protease and phosphatase inhibitors (1 mM Na3VO4, 5 mM NaF, 1mM phenylmethylsulfonyl fluoride, 10 µg/ml aprotinin, 10 µ/ml leupeptin, 10 nM okadaic acid). Cell extracts (1 mg) were pre-cleared by incubation with protein A (Prot A) at 4 ºC for 1 h (Thermo Fisher). Pre-cleared extracts were incubated with anti-PI3Kα (4 ºC; 3 h) and for 1 h more with Prot A (4 ºC). For controls, Prot A beads were incubated with extracts in the absence of antibody. IP were washed 3 times with lysis buffer, 3 times with 50 mM Tris-HCl buffer, pH 7.5 (incubated 10 min, 4 ºC) and once more with 50 mM Tris-HCl buffer, pH 7.5. The IPs were boiled in Laemmli buffer were resolved by SDS-PAGE.

### CRISPRa activation library

The human CRISPR activation library (SAM-3 system, Addgene) including 70,290 sgRNAs covering 23,430 target human genes was amplified as previously described [24].

Synergistic activation mediator (SAM) system vectors were transduced in HCC15 cells expressing inducible shRNA for *NFE2L2*. The SAM CRISPRa/dCas9 system with three vectors [24]– [25] that were efficiently transduced in HCC15 cells. Vector 1 encodes dCas9-fused toVP64 (carrying blasticidine-resistance gene) and Vector 2 encodes MS2 fused to p65 and HSF1 (carrying a hygromycin-resistance gene). Vector 3 encodes the sgRNAs (which tie up to gene promoters) fused to an RNA aptamer that binds to the MS2 protein and is selectable in puromycin. Binding of the sgRNA-MS2 to a gene promoter in cells that express the vector 1 and vector 2 results in sgRNA target gene overexpression.

SAM vectors 1 or 2 were transfected in HEK-293T cells together with psPAX2 and pCMV-VSV-G vectors. SAM vector 1 and vector 2 (Addgene) were transduced in HCC15 cells [24]. After 2 rounds of infection, one for each vector; the cells were incubated with antibiotic selection one week (blasticidin, 2 µg/ml; hygromycin, 200 µg/ml). The efficiency of the SAM system was checked using 2 sgRNAs for *CXCR4*, designed following the SAM target Golden-Gate sgRNA cloning protocol [25] in vector 3. Vector 3 encodes the lenti-sgRNA (selected with puromycin) and the RNA sequence that binds to MS2 protein (Addgene). Forward and reverse oligonucleotide sequences for the *CXCR4* sgRNAs are listed in Supplementary Table 1. Stable HCC15 clones expressing inducible shRNA for *NFE2L2* and SAM vectors 1 and 2 were infected once with the vector 3-lentiviral sgRNA (MS2)–puro CXCR4 construction. After puromycin selection (1 µg/ml, 72 h), cells were collected for qRT-PCR and PI assays. The multiplicity of infection (MOI) was set to 0.3, calculated as the number of viable cells in each condition divided by the number of viable cells in the control.

### CRISPRa library experiment workflow

To achieve sufficient representation of the sgRNA library, at least 35 × 10^6^ cells were required at the final time point [24]. HCC15 target cells (3 × 10^6^ cells/well in a 6-well plate) were seeded, infected at an MOI of 0.3, and selected with puromycin (1 µg/ml, 96 h). After one week of cell culture recovery in normal medium, 1.3 million cells/p150 plate were seeded per condition (or 0.6 million for the negative controls cells). Then, doxycycline was added (2 µg/ml;96 h) with RSL3 (4.5 µM) or DMSO vehicle for the last 24 h (Fig S4C). p150 plates were collected to extract genomic DNA. Extra plates were used to check *NFE2L2* silencing and RSL3 treatment by qRT-PCR and PI assay. A control cell plate infected with the library and without treatment was used to check the different sgRNA representation.

### NGS (next generation sequencing) and analysis

The amplified sgRNA library was sequenced to determine sgRNA distribution. To prepare the library for NGS, different forward and reverse barcode primers were used (Supplementary Table 1). Sample preparation was done as described previously [24]. Reactions were successful as they yielded a ∼270–280 bp product upon library expression. Gel-extracted samples were verified and quantified using a 2100 Bioanalyzer (high-sensitivity DNA assay, Agilent). The samples (for sgRNA distribution) were sequenced in paired-end 2 × 150 Miseq system by using MiSeq Reagent Nano Kit v2 (300 cycles) (Illumina) according to the manual. Sequencing data were analyzed in Python using the count_spacers.py script provided by the bioinformatics-genomics team of the CNB.

The remaining samples infected with the sgRNA library were sequenced similarly, with a coverage of >500 cells/sgRNA. After performing the experiment, genomic DNA was extracted from the cells for PCR amplification. Each barcoded reverse primer (with unique tags) was selected for a different culture condition (Supplementary Table 1). PCR cycling conditions were optimized as follows: 1 cycle (98 ºC, 3 min); 5 cycles (98 ºC, 10 s; 60 ºC, 10 s; 72 ºC, 25 s); 20 cycles (98 ºC, 10 s; 63 ºC, 10 s; 72 ºC, 25 s), and 1 cycle (72 ºC, 2 min). Finally, PCR products were pooled after purification. Gel-extracted samples were verified and quantified using 4200 TapeStation (D1000 assay) (Agilent Technologies) and the samples were sequenced in NextSeq 500/550 by using NextSeq 500/550 Mid Output kit v2.5 (150 cycles) (Illumina) according to the manual. Sequencing data were analyzed using MAGeCK RRA and CRISPRhieRmix software by the CNB bioinformatics team.

### SgRNAs TRIM24, PSG3, and PTGIR SAM for overexpression

sgRNAs for *TRIM24*,* PSG3*, and *PTGIR* were designed following the SAM target Golden-Gate sgRNA cloning protocol previously described and the lentiviral sgRNA (MS2)-puro backbone [24, 25]. Forward and reverse oligonucleotide sequences were designed (Supplementary Table 1). A stable HCC15 clone expressing inducible shRNA for *NFE2L2* and the SAM viral components was infected twice with lenti-sgRNA (MS2)–puro *TRIM24*, or *PSG3* or *PTGIR* constructions. After puromycin (1 µg/ml, 72 h), doxy was added at 2 µg/ml (96 h), and RSL3 (4.5 µM, 24 h). Cells were collected for qRT-PCR and PI assays.

### Subcutaneous tumor xenografts

The Ethics Committee of the CNB/CSIC approved all procedures using mice in accordance with EU/Spanish legislation (RD53/2013). For xenografts, ∼10^7^ cells of HCC15 Tet-On *NFE2L2* clone were mixed with Matrigel (at 50%, v: v) in 0.2 ml inoculated subcutaneously (s.c.) into both flanks of 8-week-old female immunodeficient athymic nude mice (Envigo) under isoflurane anesthesia. Tumors formed (3 to 7 days, with a volume of ∼100 mm^3^ ). At this point, the mice were divided into 2 groups: controls (without *NFE2L2* silencing) and a doxycycline-treated group (with *NFE2L2* silencing). Doxycycline was added to drinking water (2 mg/ml) supplemented with 5% sucrose. All animals were weighed and the volume of the tumors measured with calipers 3 times a week. Tumor volume (mm^3^) was calculated as V = (width^2^ × length)/2. We established (i) loss of 20% of initial weight or (ii) tumor size greater than 1500 mm^3^ as endpoints for the experiment.

### Statistical analysis and databases

Statistical analyses and graph generation were performed using GraphPad Prism software (GraphPad Software, Inc.) using the unpaired t-test (one- or two-tailed), chi-square (and Fisher’s exact test), or one- or two-way analysis of variance (ANOVA) (post hoc analysis selected: Tukey test). Data were represented as mean ± SD (standard deviation). The confidence interval was 95% where (*) *p* < 0.05; (**)*p* < 0.01; (***)*p* < 0.001. The databases used were the cBioPortal for cancer genomics; COSMIC and CANSAR database, and for *NFE2L2/KEAP1* alterations, the CANCERTOOL interface for tumors. David, ENRICHR, for gene ontology (GO) terms and pathways were also used. Western blot signals were measured with ImageJ software (NIH).

## Results

### NRF2/KEAP1 pathway is frequently hyperactivated in LUSC

**Fig. 1:**
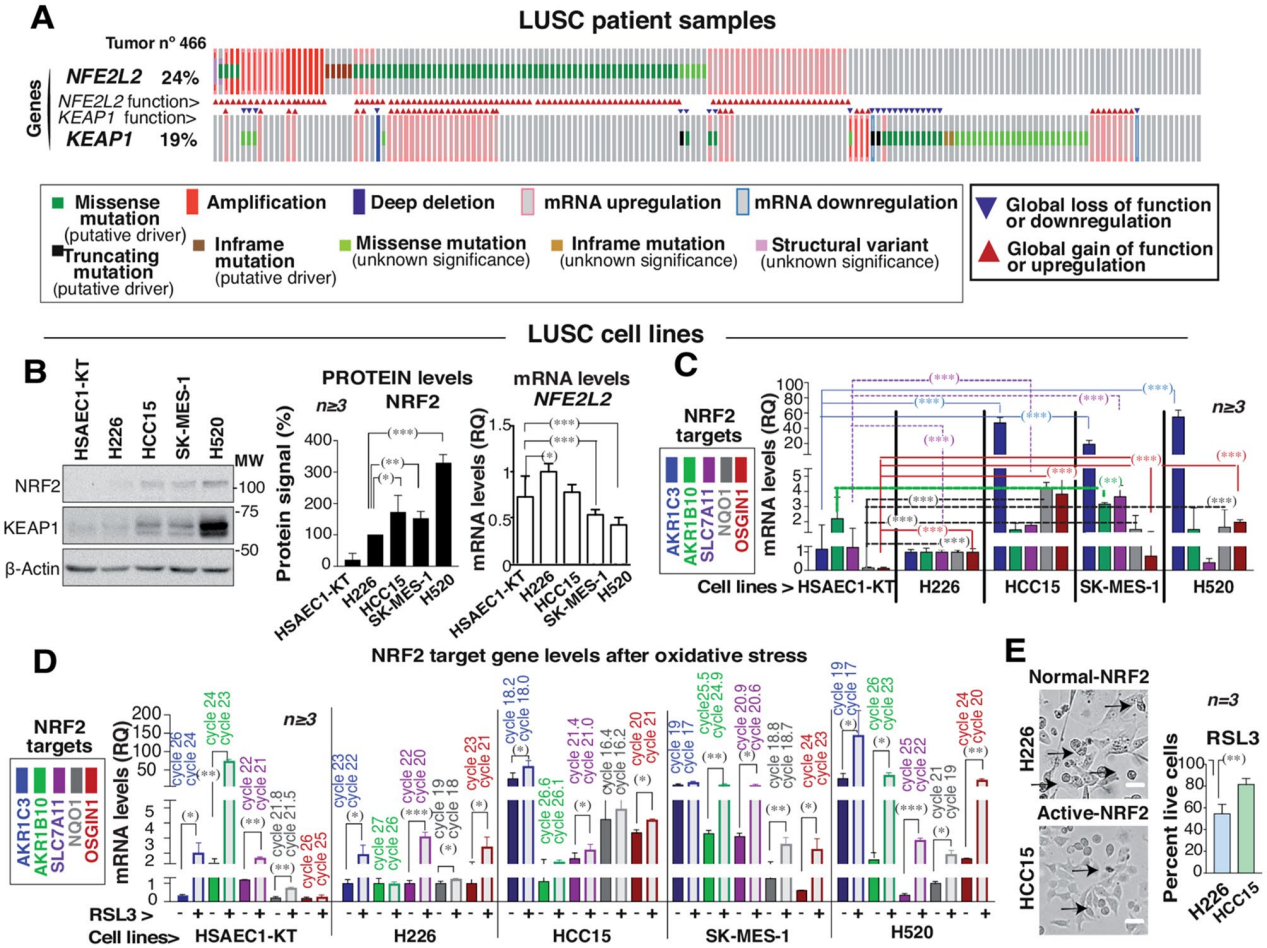
The NRF2/KEAP1 pathway is hyperactivated in LUSC. *NFE2L2* and *KEAP1* genetic alterations in LUSC patients or mRNA levels changes (www.cbioportal.com, Pan Cancer Atlas) (complete samples *n* = 466); mRNA levels of a gene is compared to its amount in diploid samples. Arrowheads indicate loss of function (blue) or gain of function (red). **(B)** A normal epithelial airway cell line (HSAEC1-KT) and several LUSC cell lines (H226, HCC15, SK-MES-1, and H520) were maintained in exponential growth, then collected for immunoblotting (indicated) or RT-qPCR. Graph showing NRF2 protein signal corrected for *β*-Actin loading and normalized to the NRF2 levels in H226 cells (100%). On the right, *NFE2L2* RT-qPCR in the different lines. Unpaired t-test (two-tailed) statistics indicated. **(C)** mRNA expression levels of several NRF2 target genes (indicated) represented as RQ values referring to those of *β-ACTIN* in H226 (considered 1). The different gene expression distribution among different cell lines was tested using unpaired t-test. **(D)** The consequences of adding oxidative stress (RSL3, 4.5 µM, 24 h) to the distinct NRF2 target´s levels in the different cell lines. Cycles number show the lower cycle value at a gene appears after RSL3 treatment. Graph as in (**C**). Unpaired t-test was used for statistics. **(E)** Normal-NRF2 (H226) and active-NRF2 (HCC15) LUSC lines were exposed to RSL3 (4.5 µM, 24 h) and collected for flow cytometry-cell viability studies using PI. Representative images after RSL3 treatment (bar: 50 μm). Black arrows indicate dead cells. The graph shows the percentage of cell viability after RSL3 treatment. Unpaired t-test for statistics. **(B-E)** Results are shown as the mean ± SD. *P* values: (*)*p* < 0.05, (**)*p* < 0.01, (***)*p* < 0.001

Gain-of-function *NFE2L2* mutations are highly prevalent in LUSC (Fig. 1A), also *KEAP1* mutations are frequently loss-of-function mutations in LUSC (http://www.cbioportal.org/). The expression of NRF2 target genes is higher in LUSC than LUAD tumors (Fig. S1A). A collection of patient-derived LUSC cell lines were analyzed to identify the status of *KEAP1* and *NFE2L2* (Fig. S1B). Among them, HCC15 cells harbor a *KEAP1* mutation (p.304G > C*)* that induces NRF2 activation (https://www.dsmz.de/collection), whereas other cells such as SK-MES-1 and H520 have an increased *NFE2L2/KEAP1* copy number ratio (https://cansar.icr.ac.uk/cansar/cell-lines/). In contrast, H226 and normal airway epithelial cells (HSAEC1/KT) harbor WT forms of *NFE2L2/KEAP1* (Fig. S1B). Other mutations in the LUSC cell lines are shown in (Fig. S1B).

NRF2 protein expression levels were lower in H226 and in HSAEC1/KT epithelial normal cells than in NRF2-active cells and those with a high *NFE2L2/KEAP1* copy number ratio (Fig. 1B). This concurs with higher mRNA levels (Fig. 1B, right bar graph). The NRF2 targets *AKR1C3*,* AKR1B10*,* SLC7A11*,* NQO1*, and *OSGIN1* were expressed in LUSC [17]. Normal HSAEC1/KT and H226 cells expressed NRF2 targets at low levels; in the active-NRF2 cells and those with a high copy number *NFE2L2/KEAP1* ratio, some NRF2 targets were expressed constitutively (Fig. 1C).

The potential coexistence of *NFE2L2* and *KEAP1* mutations in LUSC clinical samples (www.cbioportal.org/) (Fig. S1C) was examined and is not frequent. Of the six *NFE2L2/ KEAP1* double mutants detected at the same residue, one presented mutations that impair NRF2/KEAP1 complex formation in both genes; three additional locations present *KEAP1* mutations that affect the complex and a *NFE2L2* amplification or mutation (new Fig. S1C).

The overlapping of *NFE2L2* and *KEAP1* mutations with those in *TP53* or *PTEN* (frequent drivers in clinical LUSC) were examined (www.cbioportal.org/). Of the currently 487 LUSC samples in TCGA (Pan-cancer Atlas), 21% have *PTEN* mutations (around 100 patients); but only 10 samples exhibit *PTEN* and *NFE2L2* mutations and only 8 show *PTEN* and *KEAP1* co-existing mutations (Fig. S1D). As for *TP53*, this is mutated in such a high proportion of LUSC patients (86%) that *NFE2L2* or *KEAP1* end up coinciding with mutated *TP53* (Fig. S1D). The gene expression of active-NRF2 and *TP53* mutated LUSC moderately affects NRF2 target specificity [15].

Treatment with an intracellular ROS inducer RSL3, a glutathione peroxidase 4 inhibitor that increases lipid ROS, activated NRF2 and elevated its targets levels. This increase was marked in *NFE2L2/KEAP1* WT control cells (Fig. 1D), whereas in cells with a high *NFE2L2/KEAP1* copy number ratio or active-NRF2 cells (i.e., HCC15) some targets were expressed constitutively and suffer a lower change (Fig. 1C). The cycle nº in each gene is indicated in (Fig. 1D) and it is proportionally inverse to the gene levels, as low cycle nº means a sorter cycle for detection. Control HSAEC1-KT expressed *AKRB10* moderately, this could be related to the genetic background of the cells. A similar result was found upon treatment of the LUSC cells with erastin (XC- system inhibitor), which increased NRF2 target levels less markedly in H226 and HSAEC1/KT cells (Fig. S1E). When RSL3 treatment was tested for its capacity to trigger cell death, most of the HCC15 active-NRF2 cells (∼80%) survived treatment, while normal-NRF2 cells exhibited a lower cell survival (Fig. 1E). Similar results were obtained with erastin (Fig. S1F). Thus, active-NRF2 protects LUSC cells from ROS-induced cell death.

### Active-NRF2 LUSC cells lines require NRF2 for survival

To demonstrate that NRF2 acts as a driver in LUSC harboring activating *NFE2L2/KEAP1* mutations, we compared the consequences of interfering with NRF2 activity in control- and active-NRF2 cells. Initially, NRF2 inhibition was performed using the NRF2 inhibitors, AEM1 and ML385 [26, 27]. In both exponentially growing H226 and HCC15 cells, these inhibitors failed to reduce NRF2 targets levels (Fig. S2A). This was also the case upon Erastin treatment (Fig. S2A). This suggest that these inhibitors work in very specific conditions.

As an alternative approach, the effect of depleting NRF2 using a doxycycline-inducible pLKO-tet-on-sh*NFE2L2* vector was examined. HCC15 and H226 cell lines expressing inducible *NFE2L2* shRNA were prepared, and doxycycline treatment (96 h) induced a reduction of NRF2 protein and mRNA levels and its targets mRNA levels (Fig. S2B). In active-NRF2 HCC15 cells, the PCR cycles at which the target gene appeared were lower than in H226 cells (Fig. S2B), confirming the higher expression of NRF2 targets in HCC15 cells.

**Fig. 2:**
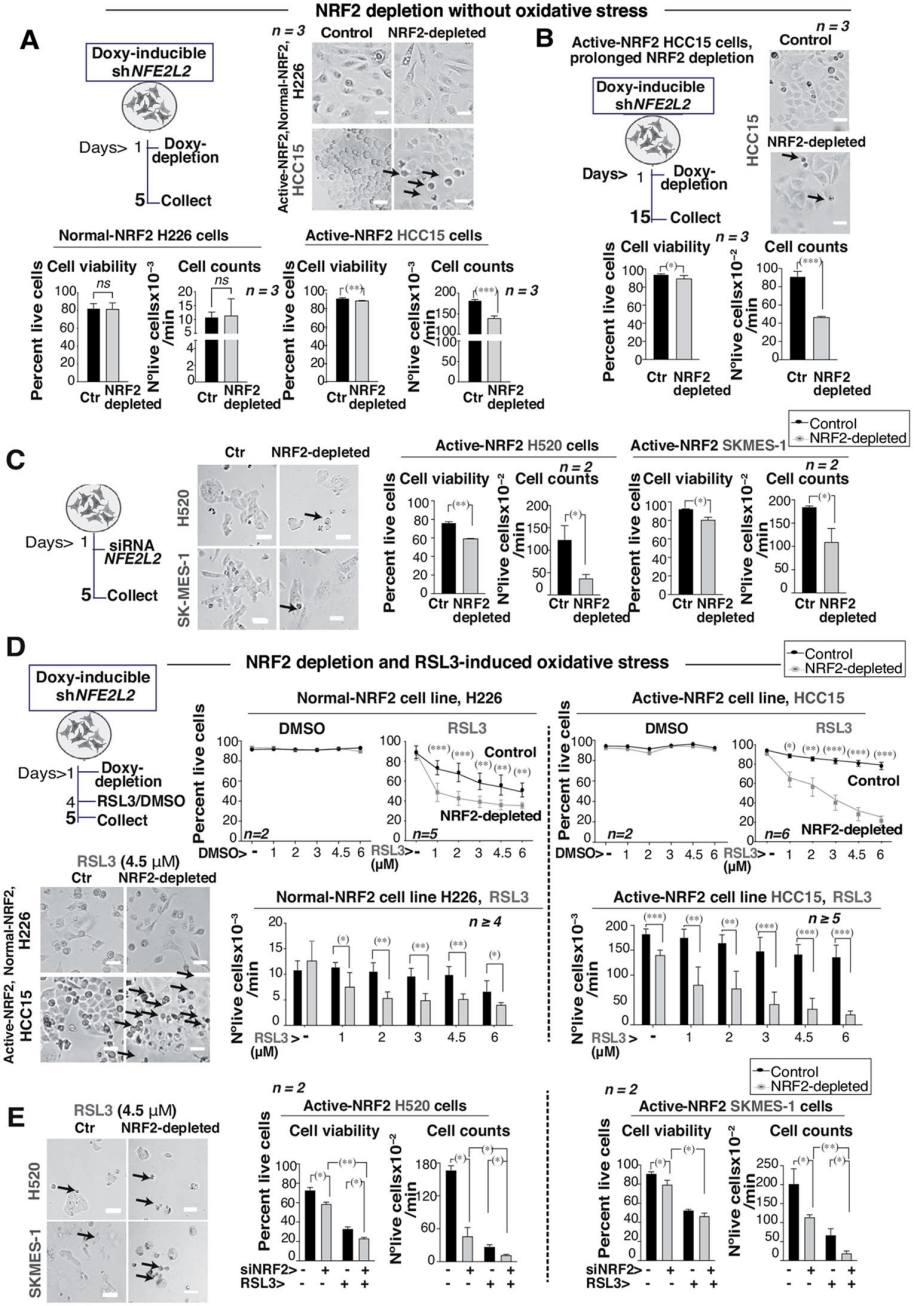
Active-NRF2 LUSC cell lines require NRF2 for survival. **(A)** Representative images of H226 and HCC15 Tet-On sh*NFE2L2* selected clones treated or not with doxycycline (2 µg/ml; 96 h) (bar: 50 μm). Graphs represent cell viability (10,000 events) and cell counting (nº viable cells/min) of propidium iodide negative cells (PI). after treatment. Unpaired t-test (two-tailed). **(B)** Tet-On sh*NFE2L2* clones from the active-NRF2 HCC15 cells were treated with doxycycline (2 µg/ml for 15 d; changed twice/wk). Then, cells were collected for cell viability analysis. Representative images (scale: 50 μm) (top); graphs show cell viability and cell counting. Statistics as in (A). **(C)** H520 and SKMES-1 were depleted of NRF2 using *NFE2L2* siRNA, examined as in (**A**). (**D)** Selected Tet-On sh*NFE2L2* clones from indicated lines were treated with doxycycline (2 µg/ml; 96 h). Then, RSL3 or its vehicle (DMSO) were added and doxycycline exchanged (24 h). The graphs represent cell viability (at day 5) (10,000 events) (top) and cell counting (bottom) after PI staining by cytofluorometry from a H226 clone or an HCC15 clone. Two-way ANOVA and Tukey test as post hoc analysis in the line graphs. In the bar graphs, unpaired t-test (two-tailed) was used for statistics. Bottom left, representative images of H226 and HCC15 shNFE2L2 clones treated with doxycycline (2 µg/ml; 96 h) and oxidative stress (RSL3, 4.5 µM, 24 h) (bar: 50 μm). A high proportion of RSL3-treated HCC15 cells exhibit a dead cell appearance (arrowhead). **(E)** H520 and SKMES-1 were treated as in (**C**) and examined as in (**A**). Unpaired t-test (two-tailed) was used for statistics. Results are shown as mean values ± SD. (*) *p* < 0.05, (**) *p* < 0.01, (***) *p* < 0.001

Cell viability was assessed in NRF2-depleted cells at day 5 (in the absence of ROS). While NRF2 depletion (96 h) did not affect H226 cell viability, it led to a reduction in both cell viability and cell count in HCC15 cells (96 h) (Fig. 2A). This effect was more evident with prolonged NRF2 depletion (15 days without ROS). This markedly reduced the number of viable HCC15 cells (Fig. 2B, Fig. S2C). Reduction of viable cells was also observed in LUSC cell lines with a high *NFE2L2/KEAP1* ratio (H520 and SK-MES-1) after NRF2 depletion using siRNA (Fig. 2C, controls in Fig. S2D).

In the presence of RSL3, normal-NRF2 H226 cells increased cell death that was proportionate to the RSL3 dose (Fig. 2D, black line). In contrast, active-NRF2 cells exhibited a higher survival rate following RSL3 treatment compared to H226 cells (Fig. 2D) (black line). When NRF2 was depleted and oxidative stress applied, the effect was more toxic for HCC15 cells than for H226 cells (Fig. 2D) (gray lines and bar graphs). Representative images of H226 and HCC15 clones treated with doxycycline (96 h) and oxidative stress (RSL3, 24 h) (bottom left) revealed a higher proportion of dead cells in RSL3-treated HCC15 compared with H226 cells (Fig. 2D). Thus, active-NRF2 cells are inherently more resistant to ROS, but when depleted of NRF2, they exhibit a higher sensitivity to ROS, suggesting that active-NRF2 cells are dependent on NRF2 for survival. H520 and SK-MES-1 cells, which reduced NRF2 upon siRNA treatment (Fig. S2F), also exhibited lower viability and lower cell number upon RSL3 treatment (Fig. 2E).

### NRF2-depletion induces cell death in LUSC PDX-derived organoids

**Fig. 3:**
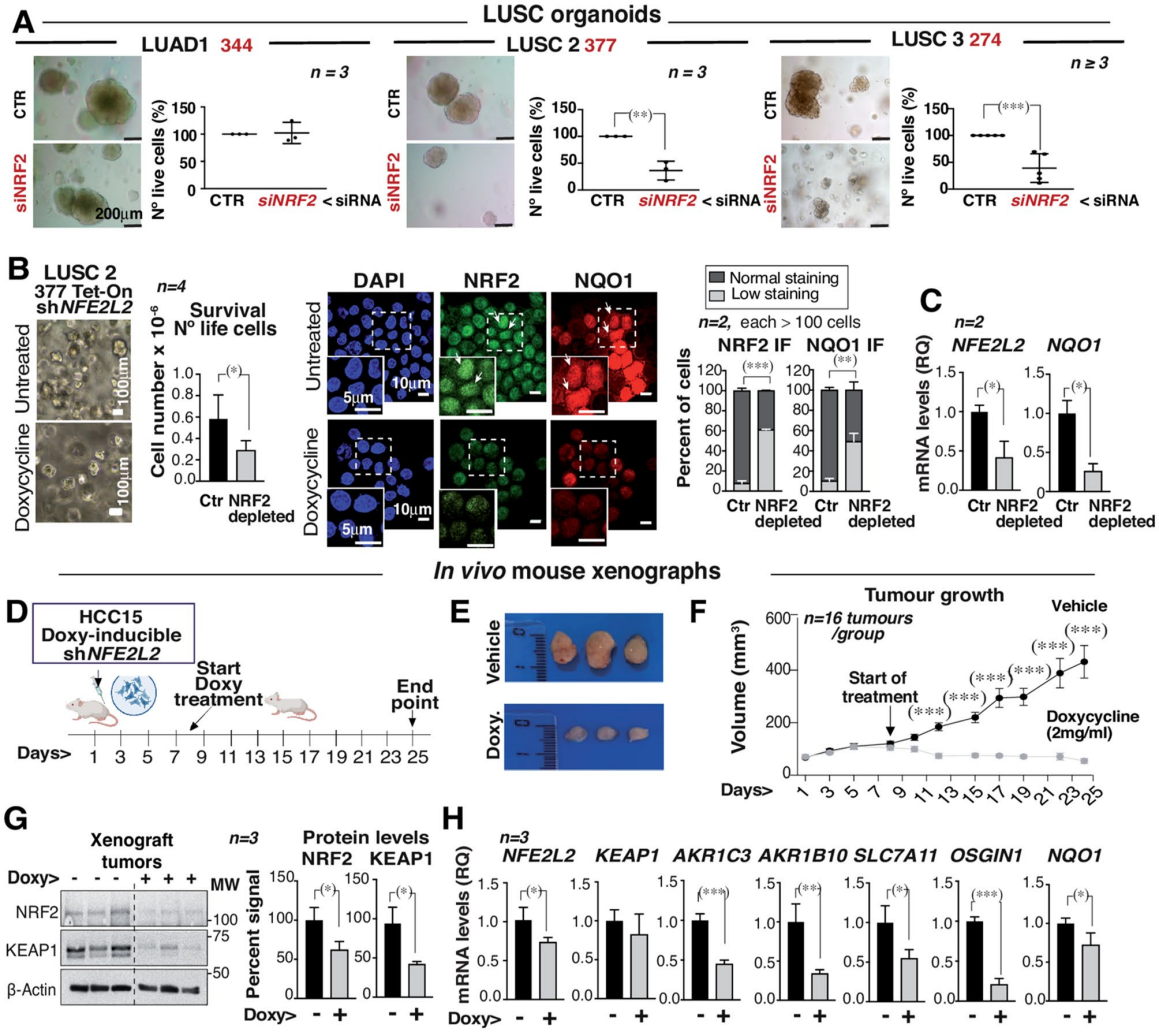
NRF2 depletion triggers cell death in LUSC PDX-derived organoids and promotes tumor regression in vivo. **(A)** The organoids were transfected with a siRNA for *NFE2L2* (96 h-1week total). Images show the different cells and the nº live cells (%) results using (propidium iodide (PI)). In (**A**) representative images (bar: 200 μm). **(B-C)** LUSC organoids were infected with lentiviral particles containing pLKO Tet-On *NFE2L2* shRNA.2. After G418 selection (0.1 mg/ml, 15 d), doxycycline was added to the culture media (2 µg/ml, 15 d; media change twice/wk). Cells were collected for number of live cells examination (in graphs). In (**A**) representative images (bar: 200 μm) **(A)**. **(B)** Representative LUSC images with cell survival numbers. In **(**B**)** IF images show LUSC 377 representative images of NRF2, NQO1 IF signals and DAPI staining, and the graphs show cell number in each condition (million cells/condition). (Bars: 5,10 μm). Arrows indicate nuclear staining. The graph at the right shows the percentage of cells with normal (> 4 × 10^3^ pixels/100 µm^2^) or low (< 2 × 10^3^ pixels/100 µm^2^) NRF2 signal. Normal (> 3 × 10^3^ pixels/100 µm^2^) or low (≤1.5 × 10^3^ pixels/100 µm^2^) NQO1 signal. Chi-Square (and Fisher’s exact test). **(C)** *NFE2L2* and *NQO1* mRNA levels in control and NRF2-depleted LUSC2 organoid represented as RQ values compared to *TBP* mRNA levels (without doxy, considered 1). Unpaired t-test. **(D-H)** HCC15 Tet-On sh*NFE2L2* selected clones were expanded and then injected subcutaneously into *scid/scid* mice (10^7^ cells in 100 µl PBS + 100 µl Matrigel). When the tumors reached ~ 100 mm^3^, the animals were randomly divided into CTR group (water) and TEST group (water with doxycycline, 2 mg/ml). Tumor size was measured 3 times/wk and at final point; tumors were collected for WB and RT-qPCR studies. **(D)** Experimental procedure for in vivo xenograft analysis (scheme). **(E)** Representative tumors. **(F)** Graph shows the size of tumors at each point (*n* = 16 tumors/group). Two-way ANOVA and Tukey test as post hoc analysis. **(G)** NRF2 and KEAP1 protein levels WB examination. Graphs show the protein signal corrected for β-Actin loading and normalized to NRF2 or KEAP1 levels in controls (100%). **(H)** RT-qPCR from extracts of HCC15 Tet-On sh*NFE2L2* selected tumors. mRNA expression levels are represented as RQ values referred to those of *GAPDH* mRNA levels in controls. Results are expressed as mean ± SD. Unpaired t-test (2-tailed). *P* values: (*) *p* < 0.05, (**) *p* < 0.01, (***) *p* < 0.001

To confirm the higher dependency on NRF2 expression in active-NRF2 LUSC cancer, alternative models were examined. An XDO from a LUAD patient (XDO344) and two from LUSC patients (XDO377; and XDO274**)** were examined [21] after NRF2 depletion. Whereas LUAD344 did not alter the number of alive cells, the two LUSC models showed reduced cell number upon NRF2 depletion (Fig. 3A; Fig. S3A controls).

Immunofluorescence (IF) staining of the LUAD1 organoid showed that the NRF2 signal was stronger in the cytosol (a sign of NRF2 lack of activity); accordingly NQO1 signal was positive in a low percent of cells (20%) (Fig. S3B). As NRF2 is not in the nucleus, RT-qPCR analysis of LUAD1 mRNA extracts showed lower expression levels of *NFE2L2*, *AKR1C3*, and *NQO1* than the expression found in LUSC2 and LUSC3 organoids (Fig. S3B, C). LUSC2 and LUSC3 cells also showed a higher NRF2 nuclear signal and a greater proportion of cells with high NQO1 IF signal content (∼50%) (Fig. S3B). Thus, LUSC2 and LUSC3 behave as active-NRF2 tumors.

Additionally, Tet-on sh*NFE2L2* was transduced in LUSC2-active NRF2 cells, and the phenotype resulting from doxycycline-induced prolonged NRF2 depletion was examined by flow cytometry. Doxycycline-treated LUSC2 organoids were smaller and the culture contained ∼50% fewer cells (Fig. 3B). NRF2 was mainly nuclear, an additional sign of NRF2 activation apart from target gene expression (Fig. 3B). Following NRF2 depletion, the remaining low NRF2 IF signal was diffusely distributed throughout the cell, and NQO1 IF intensity was markedly reduced (Fig. 3B); a small proportion of NQO1 IF signal is nuclear. *NFE2L2* and *NQO1* mRNA levels also decreased upon NRF2-depletion (Fig. 3C). The high rate of cell death observed after NRF2 depletion in active-NRF2 LUSC organoids (Fig. 3A, B), confirmed that active-NRF2 LUSC cells rely on NRF2 expression for survival.

### NRF2-depletion triggers active-NRF2 LUSC tumor regression

To determine the effects of NRF2 depletion in vivo, HCC15 cells expressing pLKO-tet-on-sh*NFE2L2* were implanted as xenografts in immunodeficient mice. When the mean xenograft size reached ∼100 mm^3^, we measured the effect of doxycycline administration in drinking water (experimental design in Fig. 3D). Doxycycline treatment resulted in tumor regression, as the size of the treated tumors was significant smaller (days 15–25) than the untreated controls (Fig. 3E, F). Tumor extracts from doxycycline-treated mice were compared with those from untreated mice, confirming NRF2 depletion and the reduction in KEAP1 protein (Fig. 3G). These tumors also exhibited a decrease in *NFE2L2* mRNA and reduced levels of the its targets (Fig. 3H). The reduction in KEAP1 protein was not detected at the mRNA (Fig. 3H). The ROS stress induced upon NRF2 depletion might trigger a reduction in KEAP1 levels [28], as ROS triggers a CUL3-dependent KEAP1 ubiquitination [7, 8]. These results confirm that NRF2 depletion induced LUSC tumor regression.

### CRISPRa/dCas9 screen define the genes that rescue survival in NRF2-depleted cells

The data described above show the dependency of LUSC cells containing *NFE2L2/KEAP1* activating mutations on NRF2 for survival. We next searched for NRF2 targets or alternative pathways that could rescue cell survival in NRF2-depleted active-NRF2 cells. These target genes likely represent either genes repressed by NRF2 (that increase upon NRF2 depletion), genes activated downstream of NRF2, or alternative cell-survival routes.

To perform this analysis, a CRISPR-activation (CRISPRa) /dead-Cas9 (dCas9) library screen was optimized. The CRISPRa/dCas9 technique takes advantage of the capacity of dCas9 to bind to a sgRNA and then to the DNA without inducing DNA cleavage. Instead, the presence of gene activation domains fused to dCas9 and MS2 enhances the expression of the genes to which the sgRNA-binds in the promoters.

**Fig. 4:**
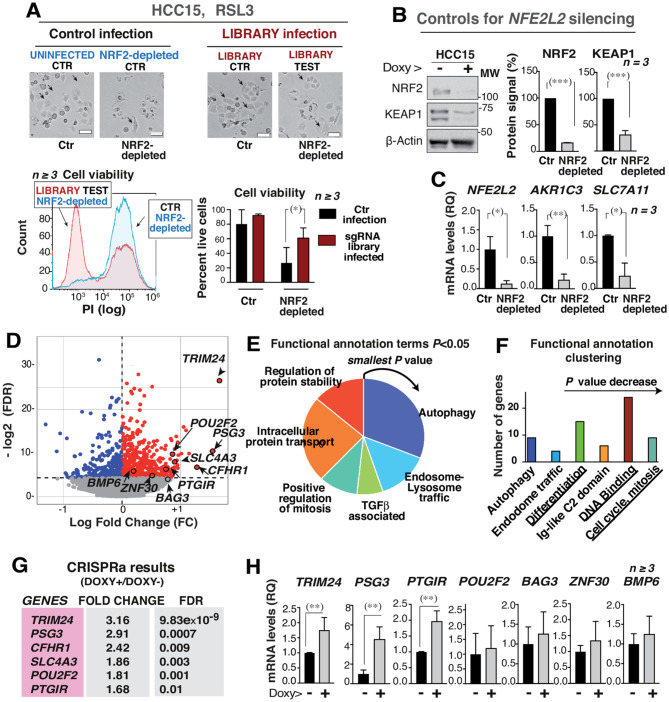
sgRNA-library infection rescues cell survival in NRF2-depleted and OS-treated LUSC cells expressing active NRF2. **(A)** Representative images of HCC15 cells (library-infected or not) and NRF2-depleted or not (scale 50 μm) (arrows indicate viable cells). The flow cytometry plots (bottom, left) show the higher mortality of NRF2-depleted cells without library infection, compared to library-infected cells. The bar graph (bottom, right) shows the percentage of PI-negative viable cells in HCC15 cells infected or not with the library (and NRF2-depleted or not). Upon doxy-induced NRF2 depletion there is a higher percentage of viable cells in the library-infected cells versus control infection. **(B)** NRF2 and KEAP1 protein levels in HCC15 cells treated or not with doxycycline. The graph shows protein signal corrected for β-Actin loading and normalized to the HCC15 control (100%). **(C)** mRNA expression levels of indicated genes from cell extracts, as in **(B)**, represented as RQ values referred to *GAPDH* mRNA levels (in CTR group). **(D)** The samples from three independent experiments were collected to compare CTR groups (sgRNA library infected and RSL3-treated HCC15 cells) and TESTs (the same but NRF2-depleted). Genomic DNA extracted and the sgRNA sequences amplified by PCR. These sequences were examined by NGS. VOLCANO plot showing the Log2 fold change of sgRNAs expression changes resulting from the NGS library analysis (x-axis) versus -Log2 (FDR) (y-axis). Some of the genes are indicated. **(E)** The round graphic shows the first functional annotation groups from David analysis (https://david.ncifcrf.gov) of the 180 genes showing an FDR < 0.05 and an increase in sgRNA levels upon NRF2 depletion. The area of each term is proportional to the number of genes (grouped by decreasing *P* values). **(F)** GO term clusters organized according to enrichment scores (x-axis) and the number of genes in the term (y-axis). **(G)** List of top genes resulting from the CRISPRa analysis. Additional criteria for gene selection was CRISPhieRmix and MaGECK analysis of gene lists, an FDR < 0.05, a change in gene levels > 1.66-fold. **(H)** mRNA levels of the selected genes in the CTR and TEST samples, represented as RQ values normalized to those of *GAPDH*. Values were referred to those in CTR group (considered 1). Mean ± SD. Unpaired t-test (one or two-tailed) (*) *p* < 0.05, (**) *p* < 0.01, (***) *p* < 0.001

The library was efficiently amplified in bacteria, as it presented the initial sgRNA clone numbers (98% sgRNAs detected and 76% sgRNAs matched perfectly). For optimization, 2 *CXCR4* sgRNA were prepared. The basal expression of CXCR4 in LUSC cells was low compared to that in Jurkat T cells (Fig. S4A). Upon co-transduction of *CXCR4* sgRNA with the library vectors 1 and 2 at an MOI of 0.3, *CXCR4* expression increased (Fig. S4B). The protocol was scaled up to ensure expression of all sgRNA in around 500 cells/sgRNA (35–36 × 10^6^ cells/assay needed). Scaled up conditions (Fig. S4C) were tested using the library plus CXCR4 sgRNA, which confirmed CXCR4 overexpression and NRF2 depletion (Fig. S4D, E). The two groups to compare were CTR (control) cells expressing vectors 1 and 2 and infected with the library, selected with puromycin, and treated with RSL3 (24 h) and TEST group, treated similarly but depleted of NRF2 (doxycycline, 4d) prior to RSL3 treatment (final 24 h). Prior to NRF2 depletion, HCC15 control cells exhibited high viability in RSL3 (> 80%) (Fig. 4A, bar graphs). This is due to constitutive expression of NRF2 targets (Fig. 4B, C). NRF2 depletion markedly reduced cell viability in HCC15 cells, and less so in library-infected HCC15 cells (Fig. 4A, bars). Survival recovery is thus linked to CRISPRa/dCas9-induced gene expression.

To define the lists of genes overexpressed, 3 experiments involving CTR and TEST samples were performed, and genomic DNA was extracted from each one. NRF2-depletion was confirmed (Fig. 4B, C). sgRNA species were amplified by PCR using 10 forward primers and a single specific reverse primer (Supplementary Table 1). The 10 PCRs for each sample were pooled, purified, and validated in a single bioanalyzer, detecting the expected ∼280 bp sgRNA product. Next generation sequencing (NGS) was performed using Illumina technology and differentiated each condition based on the reverse primers. In addition to confirming sgRNA representation in infected untreated cells (sample 5), 6 additional populations were sequenced: CTR 1-to-3, and TEST 1-to-3 samples. Both the read counts (broader in sample 5) and their distribution were examined (Fig. S4F).

Each control sample was compared with its TEST sample (Supplementary Tables 2 to 4). The different genes lists were also filtered using MaGECK and CRISPhieRmix software (only genes with a false discovery rate (FDR) < 0.05 and an expression increase > 1.50 were considered). A subsequent joint computer analysis of all samples in triplicate was performed considering both the centrality values and dispersion, not only for each experiment, but for the sgRNAs associated with each gene in each (Supplementary Table 5). Also to select a shorter list of genes, genes with the highest ratio/fold-change and a FDR < 0.01 were prioritized. A table was created to display these data by FDR or fold-change (Supplementary Tables 5,6) and visualized as a Volcano plot (Fig. 4D). Several genes were selected as HIT genes (Fig. 4D), while others that showed a poorer FDR were negative controls.

The selection of the 180 genes with an FDR < 0.05 and an increase in sgRNA levels upon NRF2 depletion was subjected to GO term enrichment analysis using the David bioinformatics resource (https://david.ncifcrf.gov/) [29]. This analysis identified key functional categories: autophagy, protein transport, cell division, and protein stability, among others (Fig. 4E).The area of each term is proportional to the number of genes. GO terms were also organized in clusters according to their gene enrichment scores (X axis) and the number of genes in the term (Y axis) (Fig. 4F). This analysis again showed cell division, DNA binding, cell cycle, etc. that could contribute to explain the phenotype.

Calculation of fold-change of gene expression changes enabled selection of the indicated genes for analysis (Fig. 4G; Supplementary Table 6). The mRNA levels of some of these HIT genes were examined in the CTR samples (with the library and RSL3 treatment) and in the library-infected, RSL3-treated, and NRF2-depleted TEST samples (1-to-3) using RT-qPCR. *TRIM24*,* PSG3*, and *PTGIR* showed a significant expression increase in NRF2-depleted cells and were the 3 hits selected (Fig. 4H). *POU2F2*,* BAG3*,* ZNF30*, and *BMP6* showed no significant increase. Expression of *SLC4A3*, which regulates cardiac potential, and *CFHR1*, associated with an atypical hemolytic-uremic syndrome, were also ruled out.

### TRIM24 is a potent regulator of cell survival in active-NRF2 LUSC cells

**Fig. 5:**
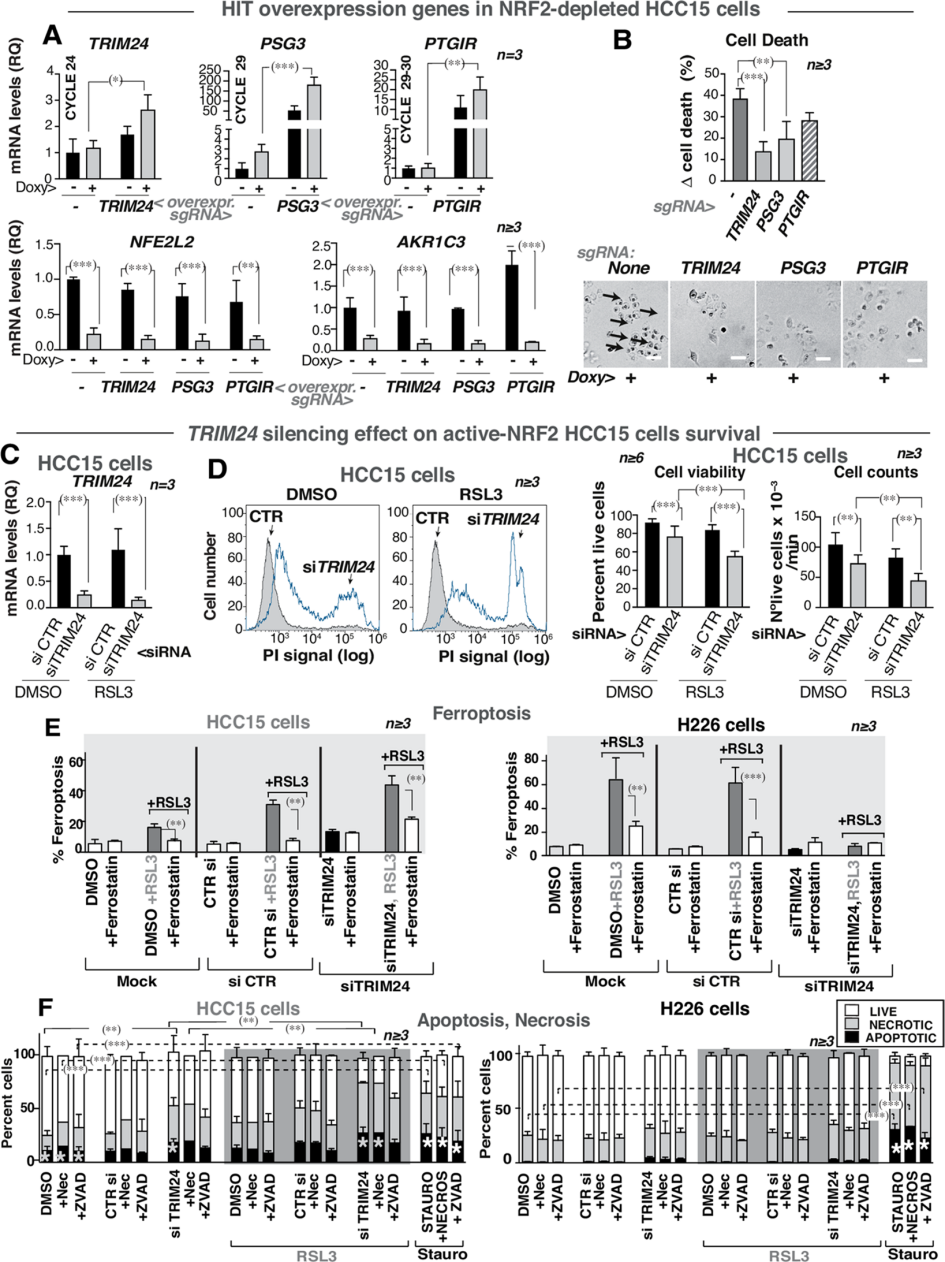
TRIM24 rescues cell survival in NRF2-depleted active-NRF2 LUSC cells. **(A-B)** Individual sgRNA for *TRIM24*,* PSG3*, and *PTGIR* genes were cloned in vector 3 and infected in HCC15 Tet-On sh*NFE2L2* cells expressing the SAM vectors. After puromycin selection (72 h), *NFE2L2* was silenced with doxycycline (2 µg/ml; 96 h) and the cells were treated with RSL3 (4.5 µM) (last 24 h). **(A)** mRNA expression levels of *TRIM24*,* PSG3*,* PTGIR*,* NFE2L2I*, and A*KR1C3*, after sgRNA lentiviral infection and NRF2 depletion. qPCRs are represented as RQ values compared to *GAPDH* mRNA and referred to control untreated cells (considered 1). Two-way ANOVA and Tukey test as post hoc analysis. **(B)** The graph shows Δ cell death representing the difference of the cell death in control cells versus that in NRF2-depleted cells, both sets treated with RSL3. Bottom: representative images of control or sgRNA infected HCC15 cells (bar 50 μm). Black arrows indicate dead cells. Statistics with Student’s *t*-test. **(C-F)** *TRIM24* double silencing was done using a specific or a control siRNA (at 24 h and 96 h), cells were treated with RSL3 (4.5 µM) or DMSO (24 h), then collected for cell different assays. **(C)** *TRIM24* mRNA expression levels after *TRIM24* siRNA transfection and treatment (DMSO or RSL3) represented and evaluated as in **(B)**. **(D)** Left, propidium iodide (PI) plots from DMSO or RSL3-treated HCC15 cells. Bar graphs (right) show cell viability and cell counting (nº of viable events/min) after indicated treatments. Statistics as in **(B)**. **(E)** H226 or HCC15 cells treated as in (**C**) were tested in a ferroptosis assay. The cells were treated with vehicle or with ferrostatin (an inhibitor of ferroptosis) (10 µM) for 2 h prior to RSL3 treatment. Bar graphs show the percentage of ferroptosis relative to total cell number (100%). Student’s *t*-test p-value. **(F)** H226 and HCC15 control or TRIM24-depleted cells were treated as in (**C**) and tested in an apoptosis/necrosis assay. Some samples were treated for the last 24 h with necrostatin (NEC, 20 µM) or ZVAD (40 µM). For apoptosis positive controls, cells were treated with staurosporin (1 µM) (24 h). Bar graphs show the percentage of viable, necrotic or apoptotic cells versus total (100%). Mean ± SD. Statistics of the comparison of percent of apoptotic cells (indicated with an asterisk) as in (**E**). Statistics with Student’s *t*-test. **(A-F)** (*)*p* < 0.05, (**)*p* < 0.01, (***)*p* < 0.001

To test whether these selected hits regulate survival, sgRNAs for the genes were prepared. Transduction of each single sgRNA increased the expression of the corresponding target, with a more pronounced effect following NRF2 depletion (Fig. 5A, top). RT-qPCR confirmed NRF2 depletion and a reduction in *AKR1C3* mRNA levels (Fig. 5A, bottom).

Among the tested sgRNAs, *PTGIR*, *PSG3*, and particularly *TRIM24* reduced cell death upon NRF2 depletion (Fig. 5B) (Δ cell death represents the difference in mortality in the absence versus the presence of NRF2, both plus RSL3). In cells lacking exogenous sgRNA, RSL3 treatment increased the death of NRF2-negative cells compared with NRF2-positive cells by ∼40%(Fig. 5B). However, *TRIM24* sgRNA transduction reduced this difference to ∼10%; *PSG3* and *PTGIR* expression reduced cell death to lesser extent (Fig. 5B).

Given that TRIM24 conferred the most robust cell-survival recovery, further analysis focused on this gene. TRIM24 was expressed at higher levels (Fig. 5A, see cycle) than PSG3 or PTGIR. Additionally, TRIM24 is known to have an epigenetic regulatory function. Whereas TRIM24 is a transcriptional coactivator, PTGIR is a prostaglandin I2 receptor and PSG3 (a pregnancy-specific β1-glycoprotein 3) that is synthesized mainly during pregnancy. Considering that TRIM24 expression increases cell survival, the consequences of its depletion were analyzed (Fig. 5C). TRIM24 depletion induced HCC15 cell death (both in the presence and absence of RSL3) (Fig. 5D).

*TRIM24* silencing was also tested in the other LUSC cell lines. All lines except normal HSAEC1 cells expressed TRIM24 protein (more in H520 cells) as well as mRNA (Fig. S4G, H). TRIM24 was silenced in the different cells (Fig. S4I).

TRIM24 depletion in normal HSAEC1/KT cells did not affect their viability; in H226 cells, TRIM24 depletion moderately reduced cell number (Fig. S5A). This might be due to TRIM24-mediated regulation of cell cycle regulators levels [30]. In cells with an altered *NFE2L2/KEAP1* copy number ratio, such as SK-MES-1, TRIM24 depletion alone had a modest effect. However, when combined with RSL3, it increased cell death and reduced the number of living cells (Fig. S5A). H520 cells were even more sensitive to RSL3 and TRIM24 depletion, as they express high TRIM24 levels (see above). The behavior of H520 and SK-MES-1 cells is similar to that of HCC15 cells in which TRIM24 depletion reduced cell viability and number in normal conditions and upon RSL3 treatment (Fig. 5D). Thus, cells with active-NRF2 forms or with an increased *NFE2L2/KEAP1* copy number ratio are more vulnerable to TRIM24 depletion than normal-NRF2 cells. In support of this view, analysis of TRIM24 alterations in patients of small cell lung cancer were at low proportion (~ 2%), whereas in NSCLC the frequency was higher (~ 12%) (www.cbioportal.org/) [19].

### TRIM24 expression rescues active-NRF2 LUSC cells from apoptosis

The mechanism of cell death regulated by TRIM24 was examined. Cells were analyzed prior to and upon transfection with either control or *TRIM24-*specific siRNA. In HCC15 cells, RSL3 induced greater ferroptosis (using C11-BODIPY) in TRIM24-depleted cells; an effect mitigated by ferrostatin 1, a ferroptosis inhibitor) (Fig. 5E). In contrast, in control-H226 cells, RSL3 induced high ferroptosis but this required TRIM24 expression (Fig. 5E, right). Thus, TRIM24 induces cell survival in active-NRF2 HCC15 cells, to some extent by restricting ferroptosis.

The effect of depleting TRIM24 on necrosis and apoptosis (using Annexin V) was tested using staurosporine as a positive control (Fig. 5F). In HCC15 cells, apoptosis was low in untreated or control siRNA transfected cells, but increased upon TRIM24 depletion or RSL3 addition (Fig. 5F, in dark). In H226 cells, apoptosis was very low in controls and RSL3-treated cells, and poorly affected by TRIM24-depletion (Fig. 5F). Examination of active caspase 3 levels confirmed apoptosis results (more in HCC15 cells) and showed that TRIM24 depletion triggers a low level of LC3B II (more in H226 cells) (Fig. S5B).Thus, in H226 cells, TRIM24 is needed for ferroptosis (Fig. 5F). In addition, TRIM24 is required in active-NRF2 LUSC cells to restrict apoptosis and ferroptosis.

### NRF2 moderately represses TRIM24 transcription

To define whether NRF2 and TRIM24 pathways are parallel or sequential, the regulation of NRF2 levels by TRIM24 (and vice versa) were examined. In active-NRF2 HCC15 cells, NRF2 depletion moderately increased *TRIM24* mRNA levels (± RSL3 treatment) (Fig. S5C) and reduced NRF2 targets levels (Fig. S5D). Immunoblot confirmed TRIM24 moderate upregulation upon NRF2 depletion, and a protein reduction by RSL3 (Fig. S5E). As for TRIM24 depletion, in active-NRF2 HCC15 cells, this did not alter *NFE2L2* mRNA levels or the levels of most of NRF2 target levels, with the exception of *AKR1C3* and *AKR1B10*, that were increased (Fig. S5G). Thus, NRF2 reduces TRIM24 levels does not alter NRF2 protein or mRNA (Fig. S5F, H), although it restricts *AKR1C3* and *AKR1B10* mRNA levels.

In H226 cells, TRIM24 did not control *NFE2L2* levels (Fig. S6A) and RSL3 increased NRF2 targets (Fig. S6B). Together, in cells with active-NRF2, NRF2 depletion moderately suppresses *TRIM24* expression, but TRIM24 does not affect *NFE2L2* levels. These findings suggest that TRIM24 is naturally upregulated when NRF2 levels decrease, supporting the role of TRIM24 expression in cell survival when NRF2 is low. The translational objective of this study is the description of HIT genes rescuing the cell death detected upon NRF2 depletion in global-active-NRF2 cells; TRIM24 achieves this objective.

### TRIM24 influences PI3K/AKT pathway activation

Active-NRF2 LUSC cells require NRF2 to survive. Without NRF2, active-NRF2 LUSC cells die unless activation of TRIM24 rescues them from cell death. To explore the mechanism responsible for TRIM24 action, we considered pathways frequently altered in LUSC that could regulate cell survival, i.e. PI3K/AKT pathway. The consequences of depleting TRIM24 on PI3K/AKT pathway activation were examined. As the PI3K/AKT pathway is negatively regulated by PTEN, and PTEN instability is mediated through MKRN1 (Makorin ring finger protein 1) [31], all these proteins were examined.

**Fig. 6:**
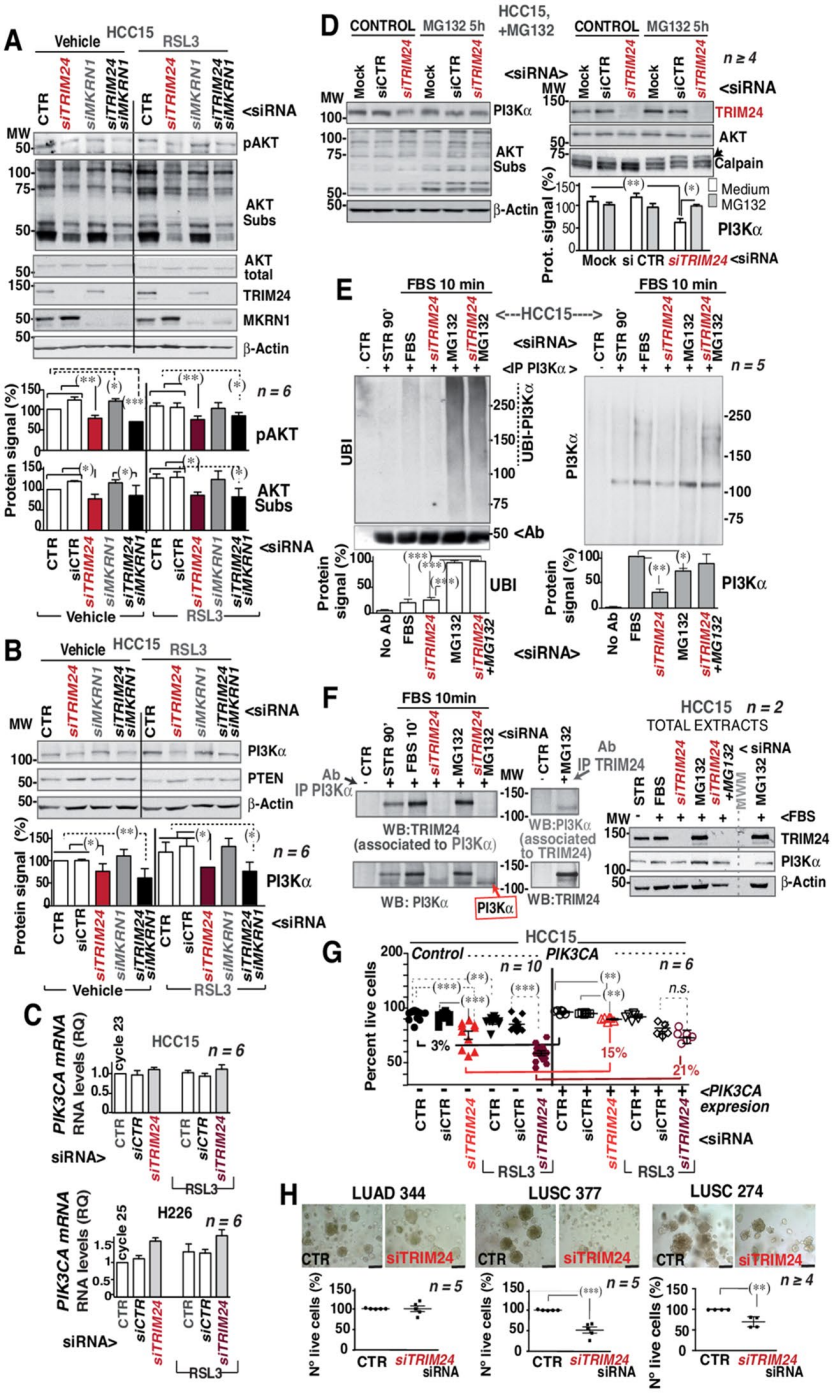
TRIM24 expression is required for PI3Kα stability and PI3K/AKT pathway activation in active-NRF2 cells. **(A-B)** Silencing of *TRIM24* and/or *MKRN1* expression was performed using control or specific siRNAs in the active-NRF2 HCC15 cell line. Two consecutive transfections were conducted (at day 1 and 4), and after 72 h cells were treated with RSL3 (4.5 µM) (24 h). Cells were collected for WB studies. **(A)** Images show the indicated WB. Graphs represent the signals corrected for β-Actin loading and normalized to the signal in CTR HCC15 cells (100%). **(B)** The images show PI3Kα and PTEN blots. The graphs show PI3Kα signal corrected for β-Actin loading and normalized to PI3Kα in HCC15 CTR cells (100%). **(C)** The graphs show *PIK3CA* mRNA levels in HCC15 cells or H226 cells depleted or not of TRIM24. RT-qPCRs were represented as RQ values compared to those of *GAPDH*, referred to untreated cells (considered 1). **(D)** Cells were treated as indicated; MG132 (10 µM) was added for the last 5 h prior to collection. Representative (indicated). The graphs show the PI3Kα signal corrected for β-Actin loading and normalized to the PI3Kα levels in mocked HCC15 cells (considered 1). An arrow points as at an upper band showing a change in mobility in calpain by MG132. Graphs as in **(A)**. **(E)** HCC15 cells were transfected twice with the indicated siRNA (see above), then cultured in medium without serum (90 min). Cells were then incubated for 10 min with 10% FBS in the presence or absence of MG132 added 5 h prior to collection. Extracts were tested by WB after PI3Kα IP. Graphs show ubiquitin and PI3Kα levels as well as Ab signal; graphs as in **(A)**. **(F)** HCC15 cells transfected or not with siTRIM24, and treated or not with MG132 (as in **E**) were collected in association buffer; the supernatants were subjected to immunoprecipitation (IP) using PI3Kα or TRIM24 Ab. Upon PI3Kα IP, the presence of associated TRIM24 was tested in WB; likewise TRIM24 IP also associated PI3Kα (as tested by PI3Kα WB). Whole extracts are showed on the right. **(G)** HCC15 cells infected with lentiviral particles containing pLV-SFFV-*PIK3CA* (7–10 days) and were sorted with GFP marker. Later, double silencing of *TRIM24* expression was performed using a control or a TRIM24 siRNA (at 24 and 96 h). Cells were treated with RSL3 (4.5 µM) (24 h). Cells were collected for cell viability analysis (propidium iodide). Student’s *t*-test used for statistics. **(H)** The organoids were transfected with siRNA for TRIM24 or control (1 week total). Images (bar: 200 μm) show the different organoids and the nº live cells (%) according to propidium iodide. Statistical analyses using unpaired *t-*test. (*)*p* < 0.05, (**)*p* < 0.01, (***) *p* < 0.001

In proliferating active-NRF2 HCC15 cells, Thr308-pAKT and AKT-substrate immunoblot signals were constitutively high and decreased after TRIM24 but not MKRN1 depletion (Fig. 6A). Treatment of HCC15 cells with RSL3 moderately increased Thr308-pAKT and AKT-substrate, which were decreased after TRIM24 depletion (Fig. 6A). Transfection of *TRIM24*-siRNA together with *MKRN1*-siRNA yielded a result similar to TRIM24 depletion (Fig. 6A), showing that TRIM24 but not MKRN1 regulates PI3K/AKT pathway in NRF2-active LUSC.

Other LUSC lines were tested, in normal-NRF2 H226 cells, TRIM24 depletion increased pAKT levels in DMSO, but not after RSL3 treatment (Fig. S6C). TRIM24 depletion did not significantly alter AKT-substrate blot patterns in H226 cells (Fig. S6C). PI3Kα or PTEN levels (Fig. S6D). However, SK-MES-1 cells (as HCC15 cells), required TRIM24 expression for optimal pAKT and AKT substrate levels (Fig. S6E). Comparison of potential correlations or the absence of them in cBio(www.cbioportal.org/) showed that *NFE2L2* and *KEAP1* mutations are mutually exclusive, and so it is *TRIM24* mutations with either *NFE2L2* or *KEAP1* mutations (Fig. S6F).

### TRIM24 regulation of PI3Kα levels in active-NRF2 LUSC

The treatment of HCC15 cells with siRNA for *TRIM24* also showed that PI3Kα levels were reduced in parallel to the AKT substrate and pAKT signals (Fig. 6B), indicating that PI3Kα levels might explain the alteration in the AKT pathway. This was not the case in normal-NRF2 H226 cells with intact PI3Kα levels (Fig. S6D).

Several hypotheses were considered to explain TRIM24 control of PI3Kα levels. The first was based on the observation of KAT6A-induced TRIM24-mediated *PIK3CA* mRNA expression in glioblastoma [20]. Examination of *PIK3CA* mRNA levels did not support a regulation by TRIM24 (Fig. 6C). Protein degradation in the lysosome or by means of proteasome action was also considered. Long serum depletion destabilizes PI3Kα via calpain activity; whereas pBRD7 and p85^PI3K^ regulatory subunit also control PI3Kα stability [32, 33]. To distinguish between these cases, cells were treated with the protein translation inhibitor cycloheximide (CHX). Whereas in controls 90 min after amino acids restoration, CHX showed good PI3Kα levels, TRIM24 depletion impaired PI3Kα levels recovery (Fig. S7A). Examination of pan-p85^PI3K^ expression showed that p85^PI3K^ subunit levels are not parallel to those of PI3Kα, suggesting that each one has its own E3-ligase (Fig. S7A).

TRIM24-depleted cells were incubated with the proteasome inhibitor MG132, which also blocks calpains. This showed that PI3Kα was stabilized by MG132 in HCC15 and not in H226 cells (Fig. 6D, Fig. S7B). The contribution of calpains for PI3Kα stability was further tested using calpain specific inhibitors [32]; these inhibitors did not alter PI3Kα in TRIM24-depleted HCC15 cells but restored PI3Kα in starving (Fig. S7C), as reported [32]. These observations show that PI3Kα stability is regulated by the proteasome.

A moderate decrease in BRD7 protein levels was detected upon TRIM24 depletion in HCC15 and H226 cells (Fig. S7D); this should render PI3Kα more stable but this is not the case. Proteasome degradation requires PI3Kα ubiquitination, it was ubiquitinated in serum activated cells and reduced following TRIM24-depletion. We found higher PI3Kα ubiquitination in MG132-treated and in MG132-treated plus TRIM24-depleted cells (Fig. 6E). This suggested that TRIM24 restricts PI3Kα ubiquitination and degradation. Thus, PI3Kα protein stability requires TRIM24 expression. The potential association of TRIM24 and PI3Kα was examined in active-NRF2 HCC15 cell extracts. PI3Kα Ab was very efficient in bringing down TRIM24 /PI3Kα complexes. The reverse experiment also show the association of endogenous TRIM24 /PI3Kα (Fig. 6F). MG132 always preserves PI3Kα stability (Fig. 6E). Nonetheless TRIM24 expression was essential for TRIM24/PI3Kα complex formation, and this complex was found in starved, in FBS-treated cells and in FBS + MG132 cells, concurring with the presence of stable PI3Kα (Fig. 6F).

### PI3Kα rescues cell death induced by TRIM24 depletion in active-NRF2 cells

To confirm the importance of PI3Kα for HCC15 cell survival, a comparison of the cell death detected in TRIM24-depleted cells plus or minus external expression of PI3Kα was examined. pLV-SFFV-*PIK3CA*-WPRE-Emerald (PI3Kα vector) was used to infect the target cells, which were sorted based on GFP levels. RT-qPCR confirmed TRIM24 depletion and PI3Kα overexpression (Fig. S7E). TRIM24 depletion in HCC15 cells reduced the percentage of viable cells more upon RSL3 addition (Fig. 6G). Whereas the percentage of control cells with or without PI3Kα overexpression varied by ~ 3%, in TRIM24-depleted cells, PI3Kα expression increased the percentage of viable cells to 15% in TRIM24-depleted and to ~ 21% in RSL3-treated cells (Fig. 6G). Very small differences in cell viability were found in H226 cells (Fig. S7F), suggesting that regulation of PI3Kα levels by TRIM24 is selective for active-NRF2 LUSC cells, in which PI3Kα restores cell survival. The potential regulation of the PI3Kα bound E3-ligases such as cCBL, CBL-b, and Anapc7 [35] was tested and discarded in (Fig. S7G).

### TRIM24 depletion induces cell death in active-NRF2 LUSC organoids

To confirm the relevance of TRIM24 in active-NRF2 LUSC tumors, TRIM24 was depleted in in organoids (Fig. S7H). TRIM24 depletion was only partial, as cells tended to remain clustered and the siRNA only reached some of them. In LUSC cells, TRIM24 depletion decreased cell survival roughly 50% (also seen in representative images), this was not the case in LUAD (Fig. 6H). Thus, TRIM24 depletion reduces cell survival in active-NRF2 LUSC organoids.

Together, NRF2 is required for active-NRF2 LUSC cells to survive (Fig. S7I). Nonetheless, when NRF2 levels are reduced, the levels of TRIM24 are increased and regulate LUSC cell survival through PI3Kα stabilization. The data supports the potential use of TRIM24-interfering compounds to destabilize PI3Kα and treat active-NRF2 LUSC patients.

## Discussion

The epithelium of squamous tissues is stratified, with proliferative basal cells residing within the squamous epithelium on the basement membrane. Basal cells migrate toward the luminal surface, undergoing terminal differentiation in the superficial compartment. The proliferation-differentiation gradient is renewed continuously and triggered by stem cells from the basal compartment. The gradient is subject to alteration during aging, infection, and malignant transformation. The normal squamous epithelium might progress to dysplasia and ultimately to LUSC, a condition associated with a very poor prognosis.

The NRF2 TF and its regulators frequently exhibit activating mutations in LUSC tumors (*NFE2L2*,* KEAP1*,* CUL3* with a combined frequency of ~ 40%). Since NRF2 regulates gene expression to counteract damage by ROS [8], it appears that LUSC is sensitive to ROS. Indeed, NRF2 depletion followed by ROS treatment causes LUSC death in the absence of expression of an appropriate HIT genes conferring survival. As NRF2 TF was difficult to block, a silencing strategy was used to bypass the NRF2 requirement and define downstream HIT targets. Thus, the main objective of this study is to identify new targets rescuing cell survival upon NRF2 depletion in active-NRF2 LUSC. To select these HITs, control and NRF2-depleted LUSC cells were infected with a CRISPRa/dCas9 sgRNA library, and genes inducing cell survival were selected. The approach defined several targets, but the focus was placed on TRIM24 (tripartite motif 24 protein) as a key gene regulating LUSC survival following NRF2 depletion (Fig. 5B). TRIM24 is a multi-domain protein that contains a RING-type E3 ubiquitin ligase domain and a terminal plant homeodomain (PHD)-bromodomain motif [34]. TRIM24, an epigenetic regulator of transcription, carries out several functions in various processes, such as cell cycle promotion [34]. TRIM24 overexpression has been associated with poor survival in cancer, in LUSC patients, and other tumors types [17, 19, 34].

Here, *TRIM24* silencing decreased NRF2-depleted active-NRF2 LUSC survival achieving the main objective. *TRIM24* silencing also reduced PI3Kα protein stability without affecting *PIK3CA* mRNA levels. PI3K is indeed frequently overexpressed in NSCLC [4, 5]. Since mRNA levels of *PIK3CA* here did not correlate with PI3Kα protein levels, this indicates a post-transcriptional regulation of expression. *TRIM24* silencing induced tumor regression in NRF2-active LUSC tumors and patient-derived organoids. In glioblastoma, *KAT6A* expression increases PI3Kα levels through the binding of TRIM24 to H3K23Ac in the *PIK3CA* gene promoter [20]. Nonetheless, *KAT6* is not often found to be increased in LUSC, although *PIK3CA* is commonly amplified (www.cbioportal.org/). As mentioned above, TRIM24 depletion did not change *PIK3CA* mRNA but it reduced PI3Kα protein levels (Fig. 6B, C). TRIM24 is known for its role in regulating transcription and might thus regulate proteins levels, factors, or pathways that permit PI3K to remain active and stable.

To examine the action of TRIM24 on PI3Kα stability, several approaches were followed. Protein degradation occurs in lysosomes and in the proteasome. Indeed, the incubation of TRIM24-depleted cells with MG132 (a proteasome and calpain inhibitor) was capable of stabilizing PI3Kα. The levels of several calpains were not altered by TRIM24, and PI3Kα stability was not altered by calpain inhibitors (Figure S7C, D), except in cases of prolonged cell starvation, when PI3Kα has been shown to be stabilized by calpains [32]. These facts suggest that PI3Kα could be a substrate for the proteasome. If this is the case, PI3Kα should undergo ubiquitination prior to degradation. Indeed, whereas PI3Kα was at low levels following prolonged starvation and this was blocked by calpain inhibitors (Figure S7C), it was overexpressed in serum-activated cells and ubiquitinated and reduced following TRIM24-depletion (Fig. 6E). MG132 corrected PI3Kα levels in TRIM24 depleted cells.

Several potential additional mechanisms involved in PI3Kα protein stabilization were examined and ruled out, including the possibility that TRIM24 alters *BRD7* mRNA levels. BRD7 binds selectively to p85 regulatory subunit, leaving PI3Kα in its monomeric form, which reduces its stability [33]. BRD7 was reduced following TRIM24 depletion, but this should stabilize PI3Kα which was indeed still unstable (Figure S7C, D).

The PHD domain of TRIM24 functions as a dual ‘reader’ of unmodified inactive H3K4 and acetylated H3K23 marks [34]. A range of functions have been attributed to TRIM24, including acting as an E3-ubiquitin ligase for p53 and associating with histone readers, estrogen, and the retinoic acid receptor [18, 34]. TRIM24 might also regulate the expression of an E3 ligase. Biogrid (https://thebiogrid.org) investigation of PI3Kα-associated proteins reveals binding of PI3Kα to E3-ligases such as cCBL, CBL-b, and Anapc7 [35], but TRIM24 did not regulated them in NRF2-active LUSC cells. As for a potential downstream regulation of mTOR, this is more frequent in adenocarcinoma (90%). Despite the remaining questions, the findings demonstrate that TRIM24 triggers PI3Kα stability by direct association in active-NRF2 LUSC cells, thereby promoting cell survival. Thus, the association of TRIM24 and PI3Kα probably protects the PI3Kα from ubiquitination/degradation, that is blocked by MG132 treatment. TRIM24-depletion triggers active-NRF2 LUSC cell death suggesting that TRIM24 represents an alternative vulnerability mechanism for active-NRF2 LUSC.

The contribution of MKRN1 to PI3Kα stability was also excluded, as its depletion did not alter PI3Kα levels. In colon cancer, p85β dissociates from the helical domain PI3K mutant, rendering it sensitive to a PI3Kα inhibitor [36]; however, this is not the case in LUSC. Together, TRIM24 depletion induces ferroptosis and apoptosis of the LUSC cells (depleted of active-NRF2); this death is probably due to reduced PI3Kα levels, as increased PI3Kα levels promoted survival.

Inhibition of PI3Kα reduces stemness marker expression in hESC and might contribute to cancer stem cell generation [37]. PI3Kβ expression is required for expression of the *NODAL* gene in hESC as well as for the TF regulating the primitive streak formation (where gastrulation begins to give rise to the 3 embryonic cell layers) [37]. Selective modulation of PI3Kα or of PI3Kβ might also be useful to treat lung tumors of different origin. Nonetheless, in the case of LUSC, SOX2 expression frequently coincides with amplifications at its chromosomal locus, and has been described as a lineage-survival factor rather than a cancer stem cell marker [38]. *PIK3CA* and *SOX2* are often amplified in the same LUSC samples [39] and TRIM24 is known to induce expression and associate with SOX2 [39]. TRIM24 might not only protect PI3Kα stability but also affect *SOX2* transcriptional activity in LUSC. *SOX2* and *PIK3CA* could promote progression by locking basal cells in a SOX2^high^ state with active PI3K signaling, which sustains the squamous injury response [39].

The presented study is limited due to the lack of additional mouse models to generate lung squamous cancer. These models require prolonged protocols (6-to-10 months), complex genetic alterations and often they render a late mixture of lung cancer phenotypes [40, 41]. Also, generation of LUSC organoids or cell lines from patients is not an easy task and they are difficult to intervene (resistant to normal transfection).

As for how to envision a potential treatment for LUSC based in these observations, these tumors may be treated with an NRF2-interfering compound. Although transcriptional inhibitors do not always yield good results (Figure S2A), dimethyl fumarate has been approved by the FDA as the first NRF2-targeting compound. Treatment of a patient with active-NRF2 LUSC using dimethyl fumarate might elicit sensitization to ROS. Since LUSC develops near the trachea and primary bronchi, upon NRF2 interference, the objective would be to increase ROS topically, with minimal effects on normal tissue. Aerosol therapy could be suitable for this treatment using metal-nanoparticles that sustain the delivery of oxidants to trigger cell death [42]. Beyond NRF2, compounds to block the HIT TRIM24-bromodomains have also been developed [43], and might impair TRIM24-induced gene expression following NRF2 interference. Alternatively, as high-TRIM24 tumors require PI3Kα expression for cell survival, PI3K specific inhibitors could also be offered to patients following NRF2 interference [44]. Other approaches involve delivery of compounds such as oridonin (in breast cancer), suppressing NRF2 and increasing apoptosis [45], or modifications of C19-position substituted geldanamycin directed to NRF2 for esophageal squamous cancer [46], or finally, direct inhibition of antioxidant systems or of TRIM24. In this regard, we await that compound 1-(indolin-1-yl) ethan-1, a TRIM24/BRPF1 bromodomain dual inhibitor, is improved before extending its use in vivo [47].

When a targeted therapy is effective, tumors tend to develop resistance mechanisms. A deeper understanding of the molecular vulnerabilities associated with targeting oncogenic-NRF2/TRIM24 will help refine the use of these compounds in LUSC. In cells with active-NRF2, NRF2 moderately represses *TRIM24* levels, but TRIM24 does not regulate *NFE2L2*. This suggests that TRIM24 is naturally upregulated gene when NRF2 levels decrease, and TRIM24 expression helps guarantee cell survival. Moreover, the druggable bromodomain of TRIM24 and its potential as a pharmacological target, highlight its significance in future therapeutic strategies for LUSC. The proportion of LUSC patients that could take advance from NRF2/TRIM24-therapies, they would be at least the ~ 40% with mutations in *NFE2L2* or *KEAP1*, as well as those tumors with *NFE2L2* high copy number or *KEAP1* low copy number. Patients tumors should be checked for the presence of increased NRF2 targets. Despite some of these mutations are silent, the patients might also have additional gene alterations in molecules (in i.e.CUL3) that affect the NRF2/TRIM24 pathway.

## Electronic supplementary material

Below is the link to the electronic supplementary material.

Supplementary Material 1

Supplementary Material 2

Supplementary Material 3

Supplementary Material 4

Supplementary Material 5

Supplementary Material 6

Supplementary Material 7

## Data Availability

The data will be deposited in Mendeley and GEO (NCBI).

## Abbreviations

ROS: Reactive Oxygen Species
NFE2L2: NFE2 Like BZIP T. Factor 2
KEAP1: Kelch-like ECH-Associated Protein1
TRIM24: Tripartite Motif Containing 24
XDO: Organoid Derived from PDX
SAM: Synergistic Activation Mediator
