# Supplementary material for: Double vulnerability of active-NRF2 lung squamous cell carcinoma to NRF2 and TRIM24
Source: Mol Cancer. 2025 Jul 17;24:197. doi: 10.1186/s12943-025-02401-y (PMC12272974; doi:10.1186/s12943-025-02401-y)
Supplement: Supplementary file 6 — Supplementary Material 6 [file 12943_2025_2401_MOESM6_ESM.pdf]

Double vulnerability of active-NRF2 Lung Squamous cell carcinoma to NRF2 and TRIM24

Miriam Sánchez-Ortega[1], Antonio Garrido[2], Lorena Sanz[1], Rafael Torres-Pérez[1], Carmen Hernandez[1], Alvaro Gutierrez-Uzquiza[3], Ming Sound Tsao[4], Ana Clara Carrera[1]*

SUPPLEMENTARY FIGURES

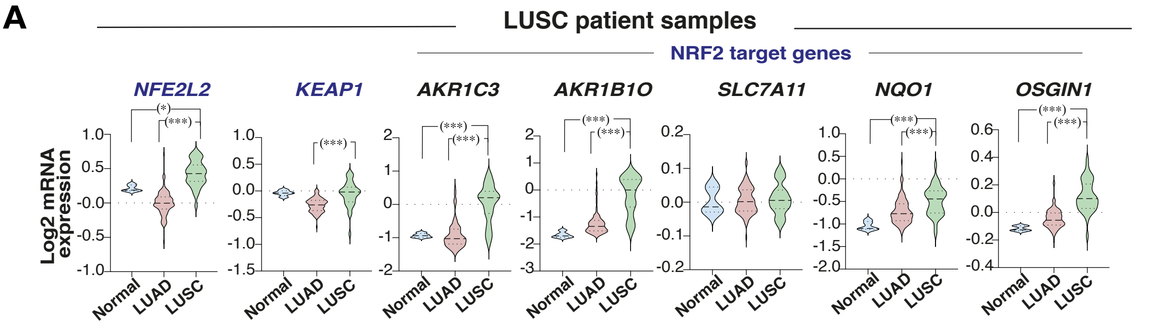

B LUSC cell lines

	Cell Line	NFE2L2 /KEAP1* mutations	KEAP1* copy nº	NFE2L2* copy nº	NFE2L2/ KEAP1 copy nº	Other genetic alterations
LUSC cell lines	HCC15	*KEAP1 p.304G>C mutation*	1	2	2	*TP53 D59V,NRasQ61K, bCatenin, PREX, Abl*, etc1
	H226	WT / WT	2	2	1	*TP53 c.473G>T, Rho GEF, APC, Kinesin*, etc1
	H520	WT / WT	4	6	1.5	*TP53 W142ns,p16, EGFR low, Rho active*, etc1
	SK-MES-1	WT / WT	2	3	1.5	*TP53 E298ns RhoGEF, cyclin E2,FK506BP1,MET* etc1
Normal cell line	HSAEC1/KT	WT / WT	2	2	1	*hTERT*

[1]https://cansar.icr.ac.uk/cansar/cell-lines/

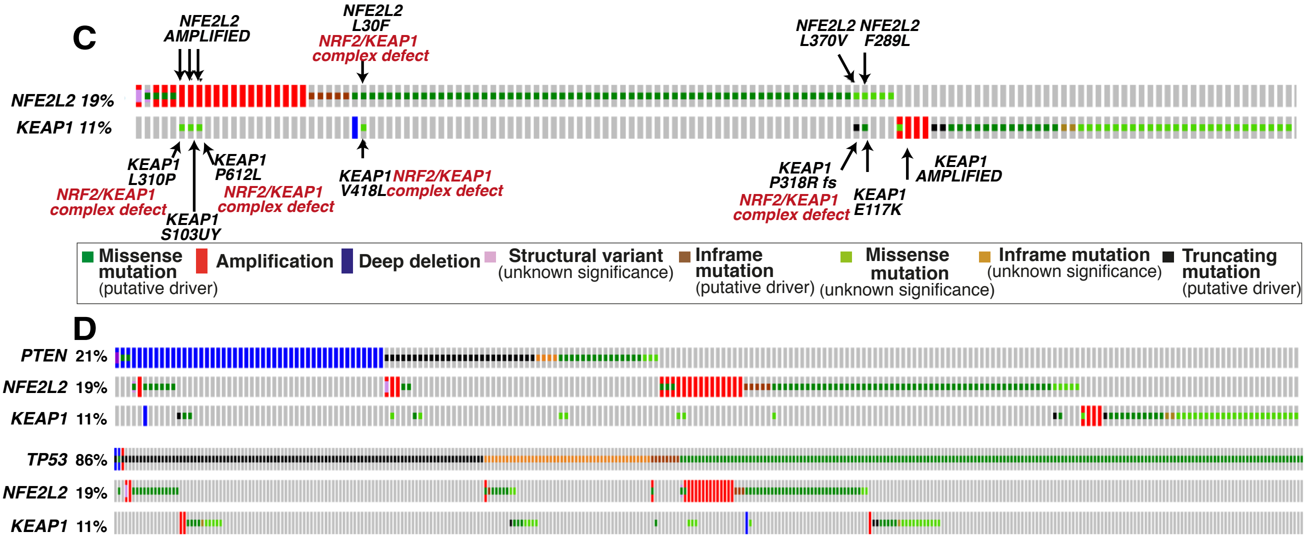

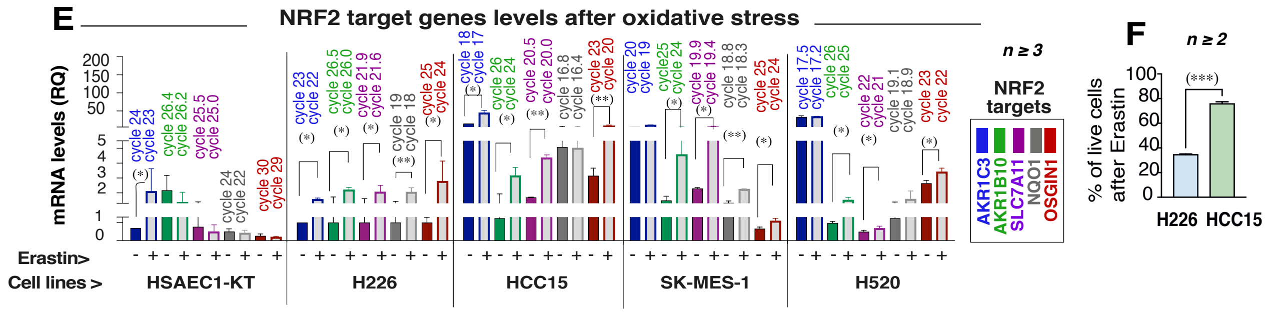

Figure 1S. NRF2/KEAP1 pathway activation in LUSC patients.
(A) mRNA expression levels of *NFE2L2*, *KEAP1* and five NRF2 target genes in normal (n = 5), LUAD (n =94) or LUSC (n = 35) tissue samples from patients. Data were from Takeuchi´s study and analyzed in www.cancertool.com. Statistics: one-way ANOVA and Tukey test as post hoc analysis. (**B**) *NFE2L2/KEAP1* genetic alterations on normal epithelial airway cell line (HSAEC1 -KT) and in four LUSC cell lines (H226, HCC15, SK-MES-1 and H520) (http://cansar.icr.ac.uk/ cansar/cell-lines). (**C, D**) Analysis of *NFE2L2* and *KEAP1* mutations and gene copy nº alteration in LUSC patients (TCGA Pan Cancer Atlas) indicating which ones affect NRF2/KEAP1 association (C) and comparison with *PTEN* or *TP53* (www.cbioportal.org/, Pan Cancer) (n=487) (D). mRNA expression of each gene compared their distribution in diploid samples. Legends as in (C). (**E**) Consequences of adding oxidative stress (Erastin, 10 μM, 24h) in NRF2 target levels in the different cell lines. Graph shows RT- qPCR analysis for the indicated genes. mRNA levels represented as RQ values normalized for β-Actin ones and referred to those of H226 cells with vehicle, considered 1. (**F**) Percent of cell viability (Erastin10 μM,24h). Mean± SD. Unpaired t-test. *P* values: (*)p<0.05, (**)p <0.01, (***) p<0.001.

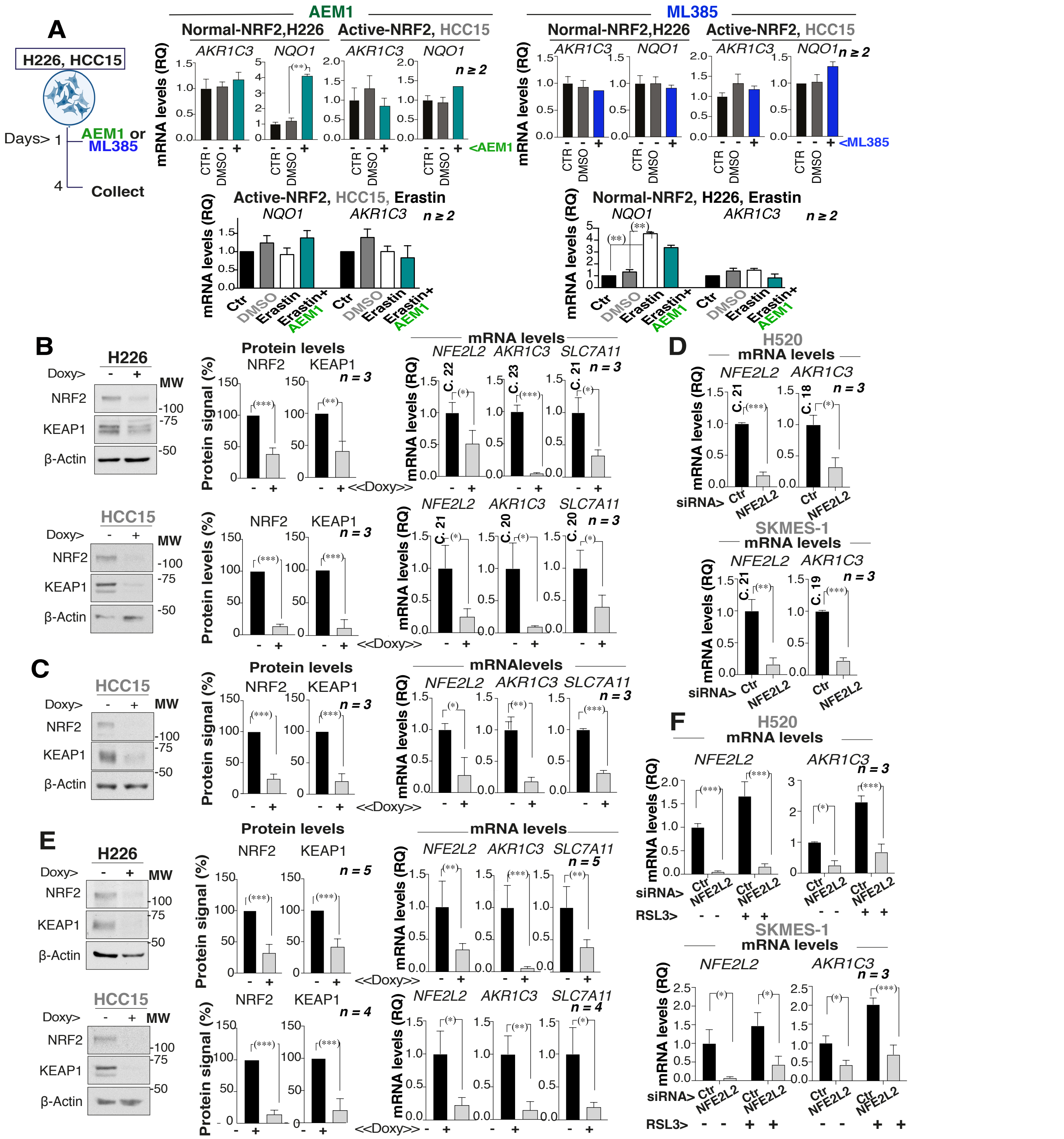

Figure 2S. NRF2 inhibitors are inactive in HCC15 and H226 LUSC lines. Controls for NRF2 depletion. **(A)** Normal-NRF2 (H226) or active-NRF2 (HCC15) cells were incubated with medium, AEM1 (10 μM) or ML385 (20 μM) NRF2 inhibitors, or their vehicle (DMSO) (72 h). Cells were collected for RT-qPCR analysis of NRF2 target genes; mRNA levels are represented as RQ (compared to *GAPDH*) and referred to untreated cells, considered 1. At the bottom , HCC15 and H226 graphs show the RQ values for *NQO1* and *AKR1C3* in cells activated with Erastin (10 μM) in the presence or not of AEM1(10 μM).(**B**) Controls for *NFE2L2* silencing (*for Fig. 2A*). Left graphs show NRF2 and KEAP1 protein signal corrected for β-Actin and normalized to NRF2 or KEAP1 levels in untreated cells (100%). Right graphs show mRNA levels of *NFE2L2, AKR1C3* and *SLC7A11*, after doxycycline treatment, represented as RQ values referred to those of *GAPDH* in untreated cells (considered 1). The qPCR cycle number (nº) in which the genes appear is indicated **(C. nº)**. (**C**) Controls for *NFE2L2* silencing (*for main Fig. 2B*) in HCC15.(**D**) Controls for *NFE2L2* silencing and *AKR1C3 mRNA* levels in H520 and SKMES-1 (*for main Fig. 2C*). (**E**) Controls for *NFE2L2* silencing (protein and mRNA) and mRNA NRF2 effectors levels in H226 and HCC15 cells (*in main Fig. 2D*). (**F**) Controls *for main Fig. 2E* in H520 and SKMES-1. Cells were NRF2-depleted and treated with DMSO or RSL3. mRNA levels of *NFE2L2* and *AKR1C3.* All the results are shown as the mean ± SD. Unpaired t-test were used for statistics. *P* values: (*) p <0.05, (**) p <0.01, (***) p<0.001.

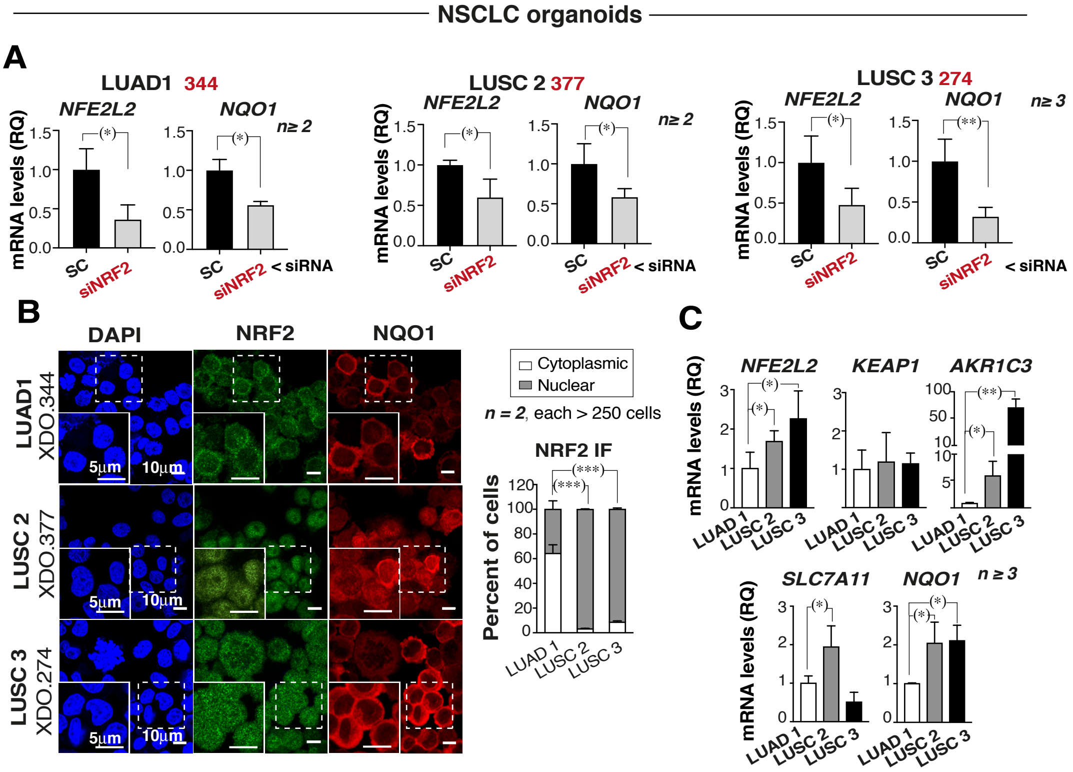

Figure 3S. NRF2 localizes in the nucleus on LUSC2 and LUSC3 organoids.
(**A**) Silencing of NRF2 gene expression was performed using a specific siRNA (96h) in 344XDO LUAD model and in the two LUSC organoids models 377XDO and 274XDO. Silencing was checked by RT-qPCR levels of *NFEE2L2 and NQO1*. **(B,C)** The LUAD organoid (XDO.344) and two LUSC organoids (377 and 274) established from cancer patients (Toronto General Hosp.) were maintained in exponential growth and collected for IF or RT-qPCR analysis. (**B**) NRF2, NQO1 and DAPI representative images. The graph in (B) represents the percentage of cells exhibiting a higher nuclear (or cytoplasmic) signal of NRF2 referred to total cell number (100%) (Scale bar 10 or 5μm). LUAD1 had NRF2 cytosolic that is a sign of NRF2 inactive. Two-way ANOVA and Tukey test as post hoc analysis. (**C**) mRNA levels of indicated genes represented as RQ values referred to those of TBP as housekeeping gene in LUAD1 XDO.344 (considered 1). Mean ± SD. Unpaired student´s t test was used for statistics. *P* values: (*) p <0.05, (**) p <0.01, (***) p<0.001.

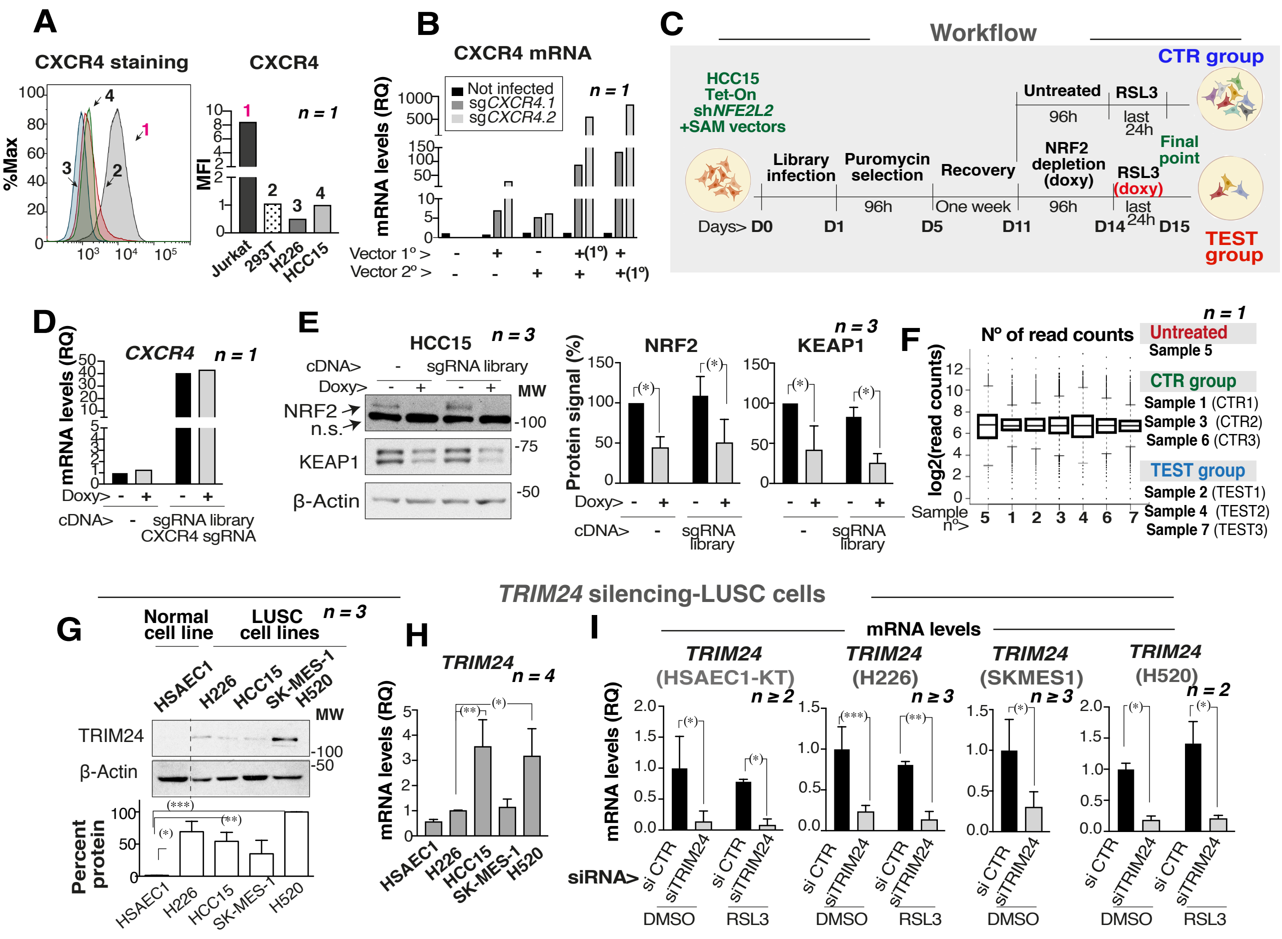

Figure 4S. Controls for the CRISPRa SAM assay used for active-NRF2 LUSC cells and controls TRIM24 depletion in LUSC cell lines.

(A) Jurkat control cells, HEK293T cells, and two LUSC cell lines were examned in flow cytometry for CXCR4 expression levels. Plot and bar graph of mean fluorescence intensity of CXCR4 staining.

(B) HCC15 Tet-On shNFE2L2 cells expressing the SAM vectors were infected with lentivirus expressing *sgCXCR4.1* or *sgCXCR4.2* cells were collected and *CXCR4* mRNA levels examined by qPCR. The graph shows *CXCR4* levels shown as RQ values (normalized to *GAPDH* in CTR, considered 1).

(C) Optimized protocol for CRISPRa library experiment. HCC15 shNFE2L2 cells expressing SAM vectors 1 and 2 were incubated at 0.3 MOI with the lentiviral particles then incubated with puromycin. After 72-96h of puromycin selection (1 μg/ml), **c**ells are kept one week without treatment and a 2º week with doxycycline to silence *NFE2L2*. Cells were treated with RSL3 or DMSO (24 h) (final point) then collected, DNA extracted, sgRNA amplified, and NGS sequenced. **(D)** CXCR4 mRNA levels at final point shown as RQ values normalized with GAPDH and referred to untreated cells. **(E)** NRF2 and KEAP1 protein analysis. For graphs the signals were quantitated, corrected for β-Actin and referred to NRF2 levels in untreated HCC15 cells (100%) Mean ± SD. Student t-test p-value (*) p<0.05.

(**F**) Representation of the number of read counts in the different NGS samples, and their frequency distribution (right).

(**G**) A normal epithelial airway cell line (HSAEC1-KT) and the indicated LUSC cell lines were grown, and collected for WB analisis. (**H**) RT-qPCR of TRIM24 mRNA levels in indicated lines. RQ values (normalized to *GAPDH* , considered 1). Statistics with student´s t-test. (**I**) TRIM24 mRNA levels in the different cells represented as RQ values normalized to those of *GAPDH*, and referred to control cells (considered 1), One-way ANOVA and Tukey test as post hoc.

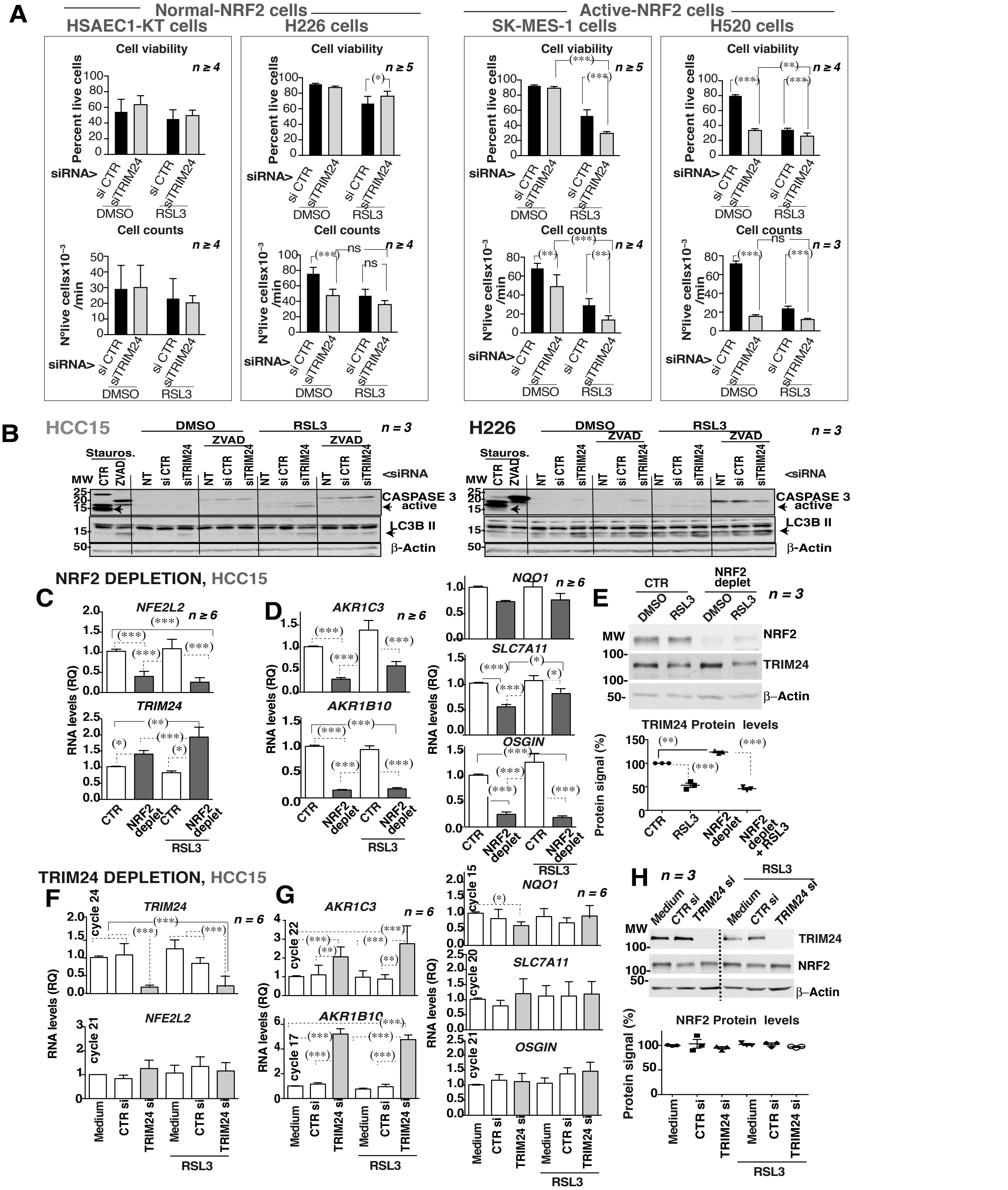

Figure 5S. TRIM24 depletion induces cell death in different LUSC cell lines and NRF2 moderately represses TRIM24. (**A**) Silencing of *TRIM24* was performed by siRNA transfection (2X) using control (CTR) or specific *NFE2L2* siRNA (72 h). Cells were treated with RSL3 (24 h). Cell viability and cell counts were examined by cytofluorometry.Mean ± SD. Statistics Student t-test p-value.(**B**) H226 and HCC15 cells were seeded and TRIM24 double silencing was done using a specific or a CTR siRNA (72 h). Cells were treated RSL3 (4.5 *μ*M)(24h) necrostatin (NEC, 20 μM) or ZVAD (40 μM). Staurosporin (1μM) was also added (24h). Cells were collected for WB (indicated).(**C-E**) Selected Tet-On shNFE2L2 clones from active-NRF2 HCC15 cells were incubated with doxycycline(2 *μ*g/ml; 72 h). Then, RSL3 was added to culture (24 h).(**C**) *TRIM24* mRNA levels after treatment with Doxyciclyne and RSL3 (2μg,ml, 96h). Data is shown as RQ values compared to those of *GAPDH*, referred to CTR cells in DMSO(taken as 1).(**D**) mRNA expression levels of several NRF2 targets represented as RQ values as in (C).(**E**) NRF2 and TRIM24 WB examination. The graph shows the protein signal corrected for β-Actin and normalized to NRF2 in HCC15 CTR cells (100%).(**F**) TRIM24 silencing (2x) was performed using a TRIM24-siRNA in active-NRF2 HCC15 cells. After 72 h, cells were treated with RSL3 (4.5 *μ*M) or DMSO (24 h) and mRNA levels examined by PCR represented as RQ values.(**G**) mRNA expression of NRF2 targets compared to *GAPDH* levels are represented as RQ values (as above). (**H**) NRF2 and TRIM24 WB examination. The graph shows the protein signal corrected for β-Actin and normalized to NRF2 in HCC15 CTRs (100%).

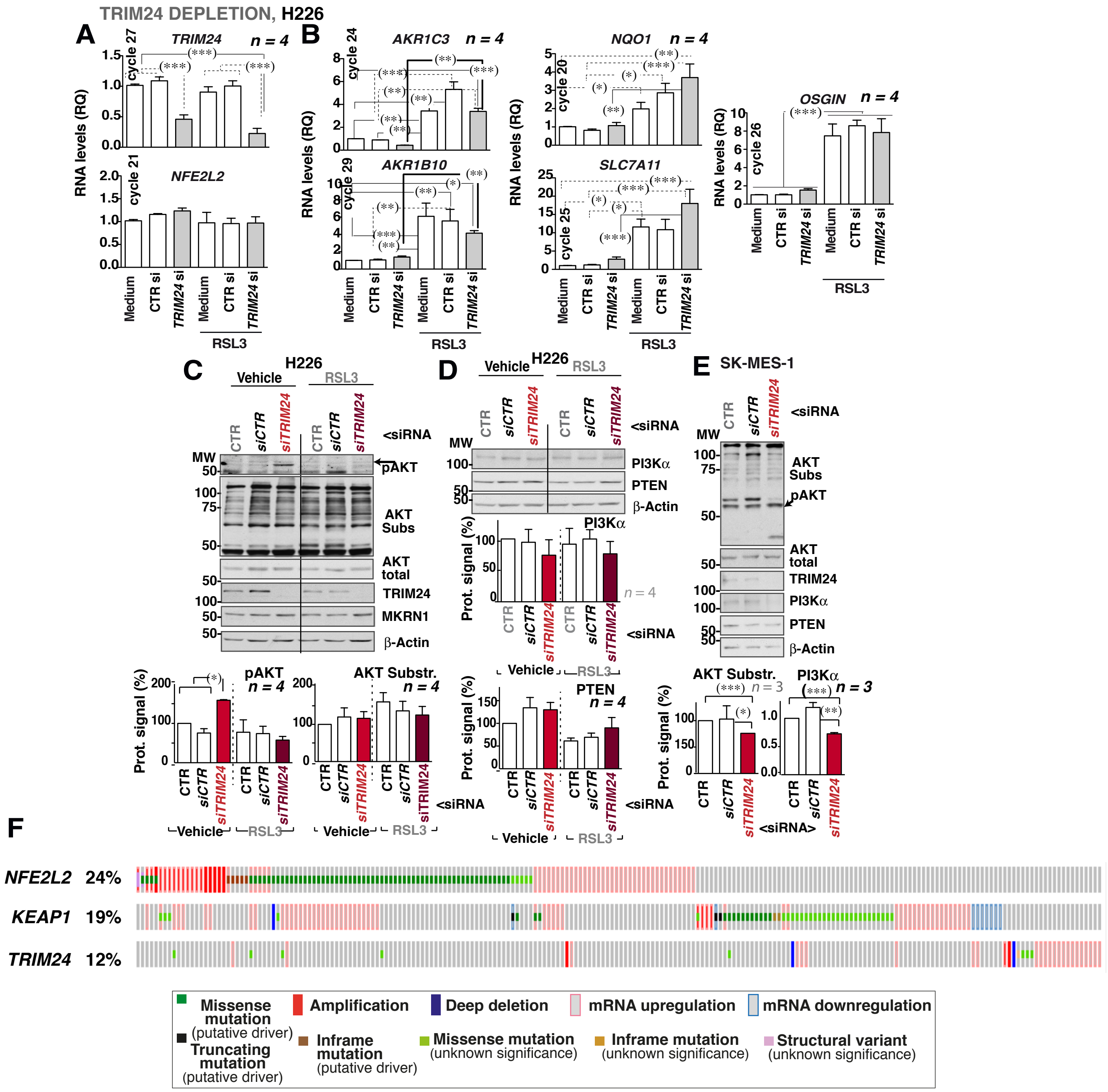

Figure S6. TRIM24 depletion does not affect PI3Kα levels in normal-NRF2 cells but it does in cells with high NFE2L2/KEAP1 copy number ratios.(A) mRNA levels of *NFE2L2* and *TRIM24* represented as RQ values and referred to *GAPDH* in untreated H226 cells.**(B)** mRNA expression levels of NRF2 target genes as in (A). All the graphs show Mean ± SD Statistics Student's t test.**(C-E)** *TRIM24* silencing was performed using siRNA in a normal-NRF2 (H226) and an in active-NRF2 LUSC line (SK-MES-1) (two transfections)(72 h), then ROS was induced by treatment with RSL3 (4.5 µM 24h). (**C**) Representative blots. The graphs shows 308pAKT, and AKT-substrate signal corrected for β-Actin and normalized to their levels in untreated H226 (considered 100). **(D)** PI3Kα and PTEN blots; graphs as in (C). **(E)** Representative blots of SKMES-1 cells showing that optimal AKT-substrate and PI3Kα signal require TRIM24 expression.*P* values: (*) p <0.05, (**) p <0.01, (***) p<0.001. **(F)** Analysis of *NFE2L2*,*KEAP1 a*nd of TRIM24 genetic alterations and change is expression in LUSC patients (www.cbioportal.org/, Pan Cancer Atlas) (n=487).

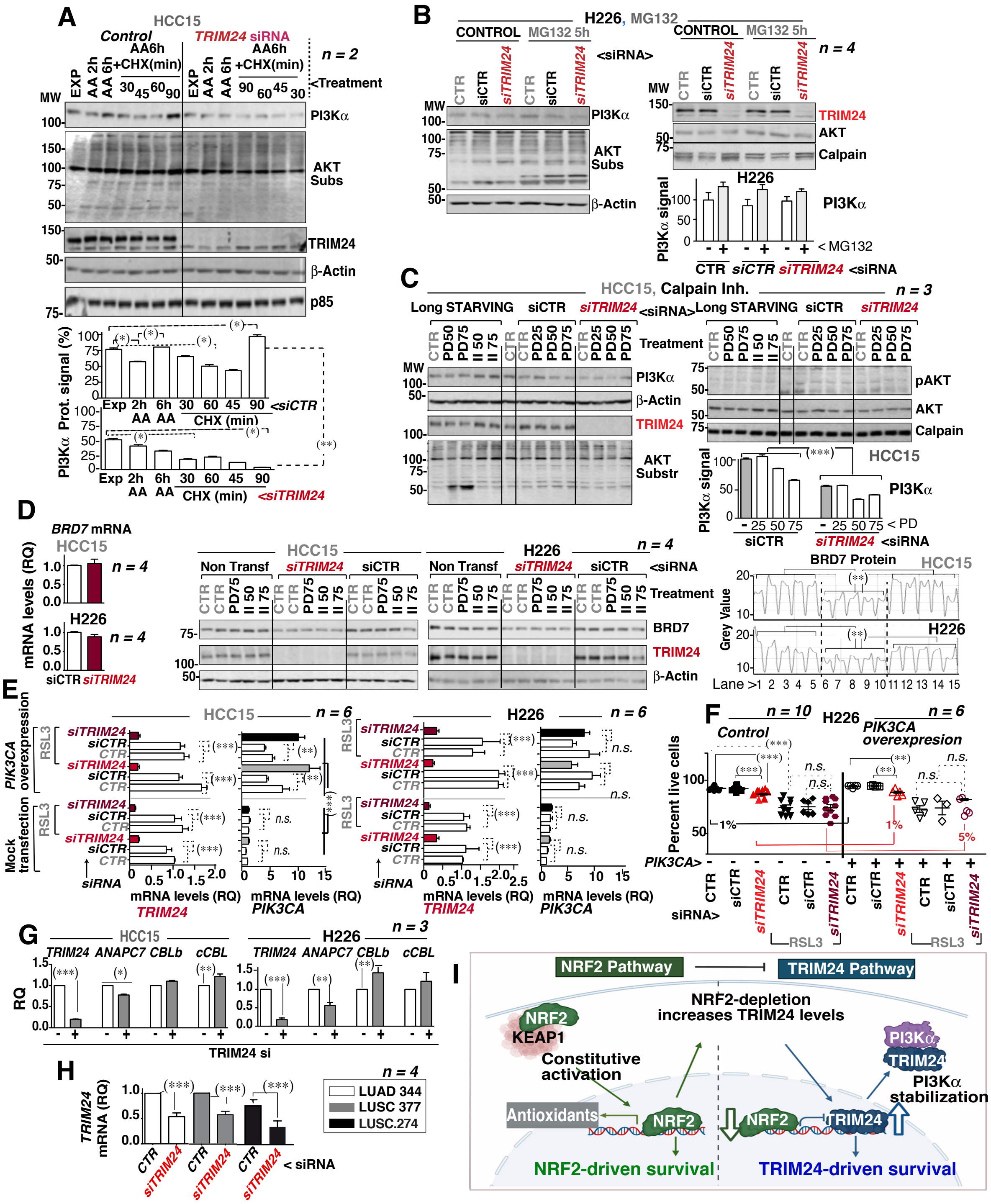

Figure S7. TRIM24 expression is not required for PI3K/AKT activation in normal-NRF2 cells.
(A) HCC15 cells were transfected with control or TRIM24 siRNA (at 24 and 72 h),then tncubated in medium without amino acids (AA) for 5 h. Then, AA were added for 2 or 6 h. After 6 h with AA, the cells were incubated with cycloheximide (CHX,half live 2h) for the indicated times, at 90min controls recovered PI3Kα and AKT signal. This did not happen with TRIM24-depleted cells. Statistics are Student´s t test. **(B)** H226 were treated as indicated, MG132 (10 μM) was added last 5h. The graph shows the PI3Kα signal corrected for β-Actin and referred to PI3Kα levels in in control H226 cells (100%). **(C)** TRIM24-depleted cells were treated with PD150606 or calpain inhibitor II (mM) for the last 16h of culture. Cells were collected an examined in WB. Graph (as in B) show that PI3Kα signal is not regulated by calpain, exccept upon long cells starving, PI3Kα increased by calpain inhibitor II. No significant difference was detected in pAKT and AKT Substrate due to the calpain inhibitors, but PI3Kα was reduced by TRIM24 depletion. **(D)** Control and TRIM24-depleted H226 or HCC15 cells were tested for RT-qPCR; other cells were also treated as indicated to test the effect on BRD7 protein abundance. The BRD7 blot was quantitated and represented in a graph showing the signal in the rectangle covering the different conditions (lanes indicated). Chi-Square (Fisher's exact test). **(E)** HCC15 and H226 cells were tranfected (or not) with siTRIM24 and infected with *PIK3CA*-Emerald particles; mRNA levels were tested in RT-qPCR as in (G). **(F)** Cell viability (30.000 events) after siTRIM24 transfection and *PIK3CA*-Emerald infection in DMSO or RSL3 treatment**.** Student´s t test was used for statistics. **(G)** mRNA levels of TRIM24, ANAPC7, CBLb and cCBL in H226 and HCC15 cells after TRIM24 depletion (*)p<0.05, (**)p<0.01, (***)p<0.001. **(H)** TRIM24 mRNA levels in the different organoids after TRIM24 depletion; Student´s t test. Graphs show Mean ± SD.**(I)** Scheme; NRF2/TRIM24 double dependence in NRF2-active LUSCs. Knockdown of NRF2 pathway results in increased TRIM24 levels. TRIM24 blocks PI3Kα degradation thereby contributing to the survival of active-NRF2 LUSC cells.
