# Supplementary material for: Double vulnerability of active-NRF2 lung squamous cell carcinoma to NRF2 and TRIM24
Source: Mol Cancer. 2025 Jul 17;24:197. doi: 10.1186/s12943-025-02401-y (PMC12272974; doi:10.1186/s12943-025-02401-y)
Supplement: Supplementary file 7 — Supplementary Material 7 [file 12943_2025_2401_MOESM7_ESM.docx]

| **NAME** | **SEQUENCE** |
| --- | --- |
| **FORWARD PRIMERS (Fwd) (5´-3´)** | |
| **NGS-Lib-SAM-Fwd-1** | AATGATACGGCGACCACCGAGATCTA CACTCTTTCCCTACACGACGCTCTTCC GATCTTAAGTAGAGGCTTTATATATCT TGTGGAAAGGACGAAACACC |
| **NGS-Lib-SAM-Fwd-2** | AATGATACGGCGACCACCGAGATCTA CACTCTTTCCCTACACGACGCTCTTCC GATCTATCATGCTTAGCTTTATATATC TTGTGGAAAGGACGAAACACC |
| **NGS-Lib-SAM-Fwd-3** | AATGATACGGCGACCACCGAGATCTA CACTCTTTCCCTACACGACGCTCTTCC GATCTGATGCACATCTGCTTTATATAT CTTGTGGAAAGGACGAAACACC |
| **NGS-Lib-SAM-Fwd-4** | AATGATACGGCGACCACCGAGATCTA CACTCTTTCCCTACACGACGCTCTTCC GATCTCGATTGCTCGACGCTTTATATA TCTTGTGGAAAGGACGAAACACC |
| **NGS-Lib-SAM-Fwd-5** | AATGATACGGCGACCACCGAGATCTA CACTCTTTCCCTACACGACGCTCTTCC GATCTTCGATAGCAATTCGCTTTATAT ATCTTGTGGAAAGGACGAAACACC |
| **NGS-Lib-SAM-Fwd-6** | AATGATACGGCGACCACCGAGATCTA CACTCTTTCCCTACACGACGCTCTTCC GATCTATCGATAGTTGCTTGCTTTATA TATCTTGTGGAAAGGACGAAACACC |
| **NGS-Lib-SAM-Fwd-7** | AATGATACGGCGACCACCGAGATCTA CACTCTTTCCCTACACGACGCTCTTCC GATCTGATCGATCCAGTTAGGCTTTAT ATATCTTGTGGAAAGGACGAAACACC |
| **NGS-Lib-SAM-Fwd-8** | AATGATACGGCGACCACCGAGATCTA CACTCTTTCCCTACACGACGCTCTTCC GATCTCGATCGATTTGAGCCTGCTTTA TATATCTTGTGGAAAGGACGAAACAC C |
| **NGS-Lib-SAM-Fwd-9** | AATGATACGGCGACCACCGAGATCTA CACTCTTTCCCTACACGACGCTCTTCC GATCTACGATCGATACACGATCGCTTT ATATATCTTGTGGAAAGGACGAAACA CC |
| **NGS-Lib-SAM-Fwd-10** | AATGATACGGCGACCACCGAGATCTA CACTCTTTCCCTACACGACGCTCTTCC GATCTTACGATCGATGGTCCAGAGCTT TATATATCTTGTGGAAAGGACGAAAC ACC |
| **REVERSE PRIMERS (Rev) (5´-3´)** | |
| **NGS-Lib-SAM-Rev-1** | CAAGCAGAAGACGGCATACGAGATTCGCCTTGGTGACTGGAGTTCAGACGTG TGCTCTTCCGATCTGCCAAGTTGATAA CGGACTAGCCTT |
| **NGS-Lib-SAM-Rev-2** | CAAGCAGAAGACGGCATACGAGATATAGCGTCGTGACTGGAGTTCAGACGTG TGCTCTTCCGATCTGCCAAGTTGATAA CGGACTAGCCTT |
| **NGS-Lib-SAM-Rev-3** | CAAGCAGAAGACGGCATACGAGATGAAGAAGTGTGACTGGAGTTCAGACGTG TGCTCTTCCGATCTGCCAAGTTGATAA CGGACTAGCCTT |
| **NGS-Lib-SAM-Rev-4** | CAAGCAGAAGACGGCATACGAGATATTCTAGGGTGACTGGAGTTCAGACGTG TGCTCTTCCGATCTGCCAAGTTGATAA CGGACTAGCCTT |
| **NGS-Lib-SAM-Rev-5** | CAAGCAGAAGACGGCATACGAGATCG TTACCAGTGACTGGAGTTCAGACGTG TGCTCTTCCGATCTGCCAAGTTGATAA CGGACTAGCCTT |
| **NGS-Lib-SAM-Rev-6** | CAAGCAGAAGACGGCATACGAGATGTCTGATGGTGACTGGAGTTCAGACGTG TGCTCTTCCGATCTGCCAAGTTGATAA CGGACTAGCCTT |
| **NGS-Lib-SAM-Rev-7** | CAAGCAGAAGACGGCATACGAGATTTACGCACGTGACTGGAGTTCAGACGTG TGCTCTTCCGATCTGCCAAGTTGATAA CGGACTAGCCTT |

**NGS forward (Fwd) and reverse (Rev) primers used for NGS.** Ten forward primers and seven reverse primers were used for the NGS study.

|  | FORWARD PRIMER (5´-3´) | REVERSE PRIMER (5´-3´) |
| --- | --- | --- |
| *sgCXCR4 1* | CACCGGCGGGTGGTCGGTAGTGAGTC | AAACGACTCACTACCGACCACCCGCC |
| *sgCXCR4 2* | CACCGGCAGACGCGAGGAAGGAGGGCGC | AAACGCGCCCTCCTTCCTCGCGTCTGCC |
| *sgTRIM24* | CACCGCGGGGAGGCAAGGGCGGGCT | AAACAGCCCGCCCTTGCCTCCCCGC |
| *sgPSG3* | CACCGACAAAGTGTGTGATGAGGCT | AAACAGCCTCATCACACACTTTGTC |
| *sgPTGIR* | CACCGGATGTCCTGGATTGGGCGGG | AAACCCCGCCCAATCCAGGACATCC |

**Forward and reverse primers used for sgRNAs SAM constructions**. Five sgRNAs for four genes (*CXCR4, TRIM24, PSG3, PTGIR*) were made using different forward and reverse primer

| GENE | FORWARD PRIMER (5´-3´) | REVERSE PRIMER (5´-3´) |
| --- | --- | --- |
| *NFE2L2* | ACACGGTCCACAGCTCATC | TGCCTCCAAAGTATGTCAATCA |
| *KEAP1* | TGGCCAAGCAAGAGGAGTTC | GGCTGATGAGGGTCACCAGTT |
| *SLC7A11* | AGCAGCAGCAGCAGTGGT | CTGTGTATGCATCGTGCTCTC |
| *AKR1C3* | CATTGGGGTGTCAAACTTCA | CCGGTTGAAATACGGATGAC |
| *AKR1B10* | AAAGCAACGTTCTTGGATGC | TGGAAGTGGCTGAAATTGG |
| *OSGIN1* | AGAAGAAGCGAAGAGGTCTTC | CCGGACACAAAGTTATGCCC |
| *NQO1* | AGGACCCTTCCGGAGTAAGA | TCCCTTGCAGAGAGTACATGG |
| *CXCR4* | GCAGCAGGTAGCAAAGTGA | GATCCCCTCCATGGTAACCG |
| *CAS9* | AAACAGCAGATTCGCCTGGA | TCATCCGCTCGATGAAGCTC |
| *MS2* | TACTCAGTTCGTGCTCGTGG | ACTAGACTGCCTGACGCTGC |
| *TRIM24* | CATATGCAGCAACAGCAACCG | GAAAGCCATCTGTAGGGGGT |
| *PSG3* | TGTCACAAAGTACACTGCAGG | AGTTGTTGATGGTGATGTAGGG |
| *POU2F2* | ACATACAGCAGCTCCTCCAGC | ATTTGGTGTCGGTAGCAGGCC |
| *BAG3* | ATGCGCGATTCCGAACTGAG | AGGATGAGCAGTCAGAGGCAG |
| *PTGIR* | TCTGCAACGGCTCGGTCACC | ATGACCACTGTCATGAGGGC |
| *ZNF30* | AGAAACCACGTGAATGTCAGG | TGTCCATTACTAAAGTTCTTCC |
| *BMP6* | ATGCAGAAGGAGATCTTGTCG | TGCTGCTGCTGCTGCTCCTC |
| *PIK3CA* | TCCAGCACATGAACGTGTAAA | TGGCTCAAAGACAAGAACAAAG |
| *PTEN* | CACACGACGGGAAGACAAGT | TCCTCTGGTCCTGGTATGAAG |
| *MKRN1* | GAAGCACCCCTGCAGGGCTCA | CTGCAGCATAGGGGCACAGCT |
| *BRD7* | AACGACGTTGGGACTTCTCC | TGCTCCATTTCTTTTGCTGTGT |
| *GAPDH* | GAAGGTGAAGGTCGGAGTC | GAAGATGGTGATGGGATTTC |
| *β-ACTIN* | GTGGGAGTGGGTGGAGGC | TCAACTGGTGTCAAGTCAGTG |
| *TBP* | TGTGCACAGGAGCCAAGAGT | ATTTTCTTGCTGCCAGTCTGG |

**qPCR primers used in the study.** Different forward and reverse qPCR primers were used for the different human genes.
